# PRSS23 promotes ovarian cancer peritoneal dissemination independent of protease activity

**DOI:** 10.1016/j.jbc.2026.111450

**Published:** 2026-04-14

**Authors:** Sharoon Akhtar, Matt Coban, Erin Miller, Alexandra Hockla, Eran Maina, Christine Mehner, Stefan Ilic, Niv Papo, Derek C. Radisky, Evette S. Radisky

**Affiliations:** 1 Department of Cancer Biology, Mayo Clinic, Jacksonville, Florida, USA; 2 Mayo Clinic Graduate School of Biomedical Sciences, Mayo Clinic, Jacksonville, Florida, USA; 3 Avram and Stella Goldstein-Goren Department of Biotechnology Engineering, Ben-Gurion University of the Negev, Beer-Sheva, Israel

**Keywords:** PRSS23, HTRA3, serine protease, pseudoenzyme, pseudoprotease, ovarian cancer, tumor metastasis, anoikis, enzyme inactivation, zymogen activation, protein secretion, protein processing, activity-based protein profiling, transcriptomics, RNA-seq

## Abstract

Ovarian cancer metastasizes *via* peritoneal dissemination, requiring tumor cells to resist detachment-induced cell death (anoikis) in suspension and to reinitiate proliferation after seeding. Because disseminating ovarian cancer cells persist in malignant ascites enriched in extracellular S1 family serine proteases, we surveyed S1 protease gene expression across ovarian cancer cohorts to identify those most associated with poor outcomes. High expression of multiple family members was associated with poor survival, with *HTRA3*, *TMPRSS12*, and *PRSS23* among the strongest hits. *TMPRSS12* transcripts were below the limit of detection in our cell line panel, while *HTRA3* depletion showed modest, histotype-dependent effects. In contrast, *PRSS23* knockdown reduced proliferation and increased anoikis sensitivity in high-grade serous and clear cell ovarian carcinoma cell lines and diminished tumor establishment, dissemination, and ascites in intraperitoneal xenograft models. RNA-seq of *PRSS23*-depleted cells revealed a conserved program of reduced cell-cycle/DNA repair gene expression with induction of inflammatory and adhesion/epithelial-mesenchymal transition pathways. Endogenous epitope tagging demonstrated that PRSS23 is synthesized as a precursor and secreted as a processed, glycosylated protease homology domain that retains the catalytic triad yet lacks the canonical Ile16-Asp194 zymogen activation switch. In complementary biochemical assays, PRSS23 showed no detectable serine hydrolase activity in either activity-based probe labeling of conditioned media or chromogenic peptide substrate assays using the recombinant protease domain. Furthermore, protumorigenic phenotypes persisted after mutation of the putative catalytic serine. Together, these findings demonstrate protease-independent PRSS23 function in ovarian cancer peritoneal dissemination and suggest that PRSS23 may ultimately warrant reclassification as a serine pseudoprotease.

Ovarian cancer remains the most lethal gynecologic malignancy, owing to late-stage presentation, high recurrence rates, and limited durability of response to current therapies (1, 2). High-grade serous ovarian carcinoma (HGSOC) is the most common and lethal form of epithelial ovarian cancer, typically presenting at an advanced stage and associated with poor clinical outcomes (2). Ovarian clear cell carcinoma (OCCC), although a rarer subtype (generally <10% of cases), is characterized by intrinsic resistance to platinum-based chemotherapy and a particularly poor prognosis when diagnosed at advanced stages (3). Clinically, ovarian cancer disseminates predominantly within the peritoneal cavity: tumor cells detach from the primary site, persist in ascitic fluid as single cells or multicellular spheroids, and seed secondary lesions on mesothelial surfaces (1, 4). This route of spread imposes two selective demands—sustained resistance to detachment-induced cell death (anoikis) during suspension and the capacity for proliferative outgrowth after seeding—that together drive recurrence and poor outcomes (5, 6). Both phases are shaped by extracellular influences, most immediately the soluble milieu of malignant ascites, enriched in growth factor and cytokine networks that influence survival, adhesion, and proliferation pathways (7, 8).

The ovarian cancer secretome, particularly in malignant ascites, is rich in extracellular enzymes and signaling mediators, with S1 family serine proteases prominently represented. Early biochemical work isolated tumor-associated trypsin(ogen)s from ovarian cyst fluid and showed that they can activate prourokinase-type plasminogen activator (uPA), placing trypsin activity at the entry point of a protease cascade relevant to tumor spread (9). Subsequent studies identified multiple serine protease systems in the ovarian cancer milieu, including the plasminogen activation axis centered on uPA (10, 11, 12), many kallikrein-related peptidases (13), cell surface-associated serine proteases such as matriptase (14), prostasin (15), and hepsin (16), and linked these enzymes to disease progression. Through matrix remodeling, growth factor and cytokine processing, and receptor/adhesion regulation, these proteases can alter the cues that govern detachment survival, mesothelial infiltration, and expansion at peritoneal sites (17). In line with these observations, activity-based profiling of ovarian cancer secretomes identified uPA along with tissue-type plasminogen activator as functional drivers in OCCC, while gene silencing of either suppressed invasion and proliferation (18). Notably, the S1 family is the largest and most diverse protease family in humans, comprising over 100 secreted or membrane-tethered enzymes (18, 19), yet only a subset has been investigated in ovarian cancer, highlighting the need to define which S1 serine proteases are most strongly associated with clinical progression.

There are compelling biological and translational reasons to evaluate S1 serine proteases broadly in ovarian cancer. First, proteases act within interconnected networks; trypsin-like enzymes can initiate or amplify cascades (for example, conversion of prourokinase to uPA), and redundancy and cross-activation can create parallel routes to the same malignant phenotypes (17, 20). Focusing on just a few canonical nodes risks overlooking alternative control points that tumors may exploit within the peritoneal milieu. Second, histotype differences in genetics, microenvironmental context, and therapy response suggest value in comparing subtypes such as HGSOC and OCCC (2, 3); while some protease dependencies may be shared, others may reflect distinctions between subtypes. Third, the vast majority of S1 serine proteases (>97%) are extracellular secreted or cell surface-associated enzymes (18), making them accessible and actionable as targets for pharmacologic inhibition (21), antibody targeting, and biomarker development in fluids such as ascites. Guided by these considerations, we undertook a broader evaluation of the S1 family in ovarian cancer.

Here, we performed a systematic, outcome-based analysis of public ovarian cancer cohorts to identify S1 serine proteases with the strongest and most consistent associations with adverse clinical outcomes for subsequent mechanistic study. In this survey, expression of many S1 family members was linked to worse overall survival (OS). Among the most strongly associated were *HTRA3* and *PRSS23*, encoding proteases previously little-studied in ovarian cancer, which we then prioritized for functional studies in representative HGSOC and OCCC models to define their potential roles in detachment survival and growth. PRSS23 emerged as a mediator of both anoikis resistance and proliferation across subtypes, with *in vivo* corroboration of reduced peritoneal tumor burden upon its loss. Despite secretion of its mature serine protease homology domain, the effects of PRSS23 on ovarian cancer cells did not require proteolytic activity, demonstrating a nonproteolytic mode of action.

## Results

### Expression of multiple S1 serine protease genes is associated with poor survival in ovarian cancer

To systematically survey the clinical relevance of the S1 serine protease family in ovarian cancer, we first used the curated MEROPS Peptidase Database (19) to compile all human S1 peptidase domains annotated as known or putatively active based on conservation of the Ser-His-Asp catalytic triad. From an initial list of 119 entries, exclusion of six lacking gene annotations yielded 113 peptidase domains mapping to 111 unique genes. Of these, 93 genes were represented in the KMplotter platform, which integrates transcriptomic data with survival outcomes across multiple cancer datasets (22). We analyzed association of these 93 genes with OS in ovarian cancer using hazard ratios (HRs) and log-rank *p* values (Table S1). Among these genes, 32 showed HR > 1, with 15 significantly associated with worse OS (*p*< 0.05). The top eight genes by OS HR are summarized in Table 1, with *HTRA3*, *TMPRSS12*, *PRSS23*, and *PLAU* exhibiting the highest HRs, ranging from 1.58 to 1.34. Notably, *PLAU*, encoding uPA, a well-established mediator of ovarian cancer dissemination and poor outcome (10, 11, 12, 18), ranked among the strongest hits in this survey (Table 1; ranked fourth by OS HR). To further contextualize the magnitude of these HRs, we applied the same KMplotter analysis to a small set of genes previously reported as ovarian cancer prognostic markers; these comparator genes generally exhibited slightly more modest OS HRs than the top-ranked S1 candidates (Table S2). Taken together, the magnitude of the OS HRs and the emergence of *PLAU* as a top hit provide an internal point of reference that our outcomes-based approach recapitulates known clinically relevant protease biology, while highlighting *HTRA3*, *TMPRSS12*, and *PRSS23* as comparably strong or stronger survival-associated candidates within the S1 family.

**Table 1:** Top S1-family serine protease genes associated with poor overall survival in ovarian cancer

#	MEROPS ID	Peptidase	Gene	HR (OS)^a^	*p* value^b^
1	S01.284	HtrA3 peptidase	*HTRA3*	1.58	4.70E-05
2	S01.291	TMPRSS12 peptidase	*TMPRSS12*	1.49	2.60E-04
3	S01.309	Serine protease 23	*PRSS23*	1.43	5.80E-04
4	S01.231	Urokinase-type plasminogen activator	*PLAU*	1.34	4.10E-05
5	S01.299	PRSS55 g.p. (*Homo sapiens*)	*PRSS55*	1.33	1.00E-02
6	S01.365	TMPRSS11B peptidase	*TMPRSS11B*	1.33	9.90E-03
7	S01.251	Kallikrein-related peptidase 4	*KLK4*	1.32	1.20E-02
8	S01.286	Tysnd1 peptidase	*TYSND1*	1.29	3.40E-02

a HR (OS), hazard ratio for overall survival; HR > 1 indicates worse OS in the high-expression group.

b Log-rank *p* values.

**Figure 1:**
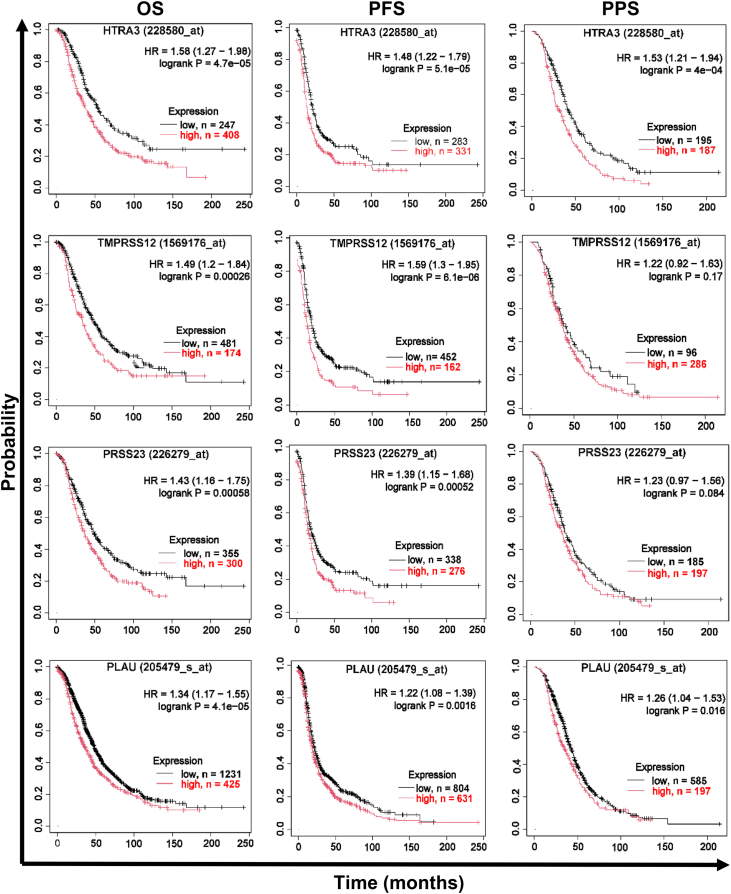
**High expression of selected S1 family serine proteases is associated with poor outcome in ovarian cancer.** Kaplan–Meier plots illustrate overall survival (OS), progression-free survival (PFS), and postprogression survival (PPS) for ovarian cancer patients stratified by high (*red*) *versus* low (*black*) gene expression of the *top* four S1 family serine proteases by OS hazard ratio (HR): *HTRA3*, *TMPRSS12*, *PRSS23*, and *PLAU* (top to bottom). Statistical analysis was performed by the KMplotter platform, with log-rank *p*-values and HRs indicated on each plot.

Kaplan–Meier plots for these top four genes (Fig. 1) illustrate stratification across OS, progression-free survival (PFS), and postprogression survival. *HTRA3* (high-temperature requirement A serine peptidase 3), encoding an active, secreted serine protease with roles in placental development (23) and reported effects on apoptosis and therapy response in lung cancer (24, 25), shows robust associations across all three ovarian cancer endpoints, with higher *HTRA3* expression associated with worse outcomes (Fig. 1). *TMPRSS12* (transmembrane protease, serine 12), encoding an active type-II transmembrane serine protease with essential roles in male fertility (26, 27) and little prior research in cancer, is strongly associated with poor ovarian cancer OS and PFS here, with higher *TMPRSS12* expression associated with worse outcomes (Fig. 1). *PRSS23* (serine protease 23) is predicted by homology to encode an active protease domain, but its putative activity has not previously been characterized biochemically. It has been implicated previously in cardiac valve morphogenesis (28) and in several other cancer settings (29); in the present analysis, it strongly associates with poor OS and PFS in ovarian cancer cohorts (Fig. 1). *PLAU* (uPA) also associates with poor clinical outcomes across all three endpoints (Fig. 1). Together, these analyses indicate that elevated transcript expression of multiple S1 serine proteases correlates with adverse outcomes, with endpoint-specific differences likely reflecting both biology and cohort sizes.

Because the top three genes—*HTRA3*, *PRSS23*, and *TMPRSS12*—exhibited large HRs with strong statistical support yet remain comparatively understudied as functional drivers in ovarian cancer, we next asked whether they are expressed in models suitable for mechanistic interrogation. Baseline profiling across 13 ovarian cancer cell lines spanning HGSOC and OCCC histotypes, aggressive subtypes with poor outcomes and limited durable therapies (30), showed that *HTRA3* and *PRSS23* transcripts were reliably detectable across multiple cell lines in our panel (Fig. S1), whereas *TMPRSS12* was below the limit of reliable detection in all cell lines. We therefore next advanced *HTRA3* and *PRSS23* for genetic perturbation and phenotypic analysis in representative HGSOC and OCCC models.

### HTRA3 knockdown reveals subtype-dependent effects on proliferation and anoikis in ovarian cancer cells

**Figure 2:**
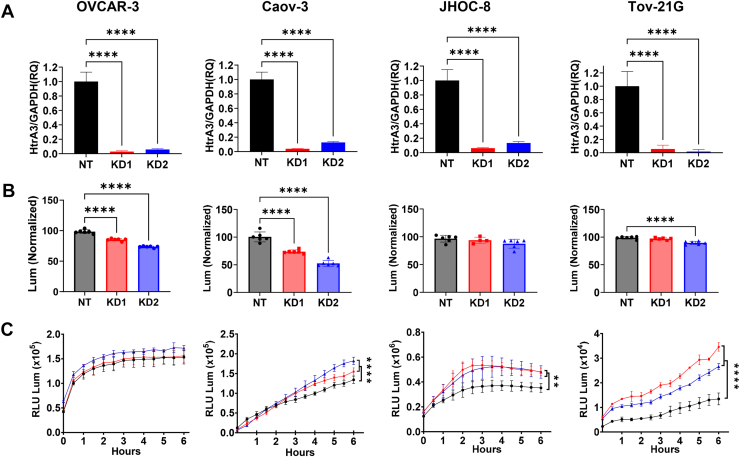
***HTRA3* depletion produces histotype-dependent effects on ovarian cancer proliferation with modest effects on anoikis.***A*, validation of *HTRA3* transcript knockdown in ovarian cancer cell lines following lentiviral transduction with two independent shRNAs (KD1, *red*; KD2, *blue*) compared to nontargeting control (NT, *black*). Cell lines shown: OVCAR-3 and Caov-3 (HGSOC) and JHOC-8 and TOV-21G (OCCC). Expression levels were normalized to *GAPDH* and analyzed by qRT-PCR; plots show mean ± SEM. N = 3 per condition. *B*, quantification of proliferation using luminescence-based CellTiter-Glo assays. Data represent percentage proliferation over 16 h relative to NT. N = 6 per condition. *C*, detachment-induced apoptosis under low attachment conditions measured over time using the RealTime-Glo Annexin V apoptosis assay; increased luminescence reflects increased Annexin V signal (phosphatidylserine exposure), consistent with anoikis. N = 6 per condition. Data are presented as mean ± SD with individual data points superimposed. Statistical analysis was performed using one-way ANOVA with Dunnett’s multiple comparisons test for (*A* and *B*) and two-way ANOVA with Dunnett’s multiple comparisons test for (*C*); ∗∗*p*< 0.01, ∗∗∗∗*p*< 0.0001. HGSOC, high-grade serous ovarian carcinoma; OCCC, ovarian clear cell carcinoma.

Because ovarian dissemination imposes selective demands of (*i*) resistance to detachment-induced cell death (anoikis) and (*ii*) proliferative outgrowth, we next tested whether *HTRA3* contributes to these traits. Two independent short-hairpin RNAs (shRNAs) were used to deplete *HTRA3* in OVCAR-3 and Caov-3 (HGSOC) and JHOC-8 and TOV-21G (OCCC); quantitative reverse transcription polymerase chain reaction (qRT-PCR) confirmed efficient silencing in all four lines (Fig. 2*A*). *HTRA3* knockdown significantly reduced proliferation in both HGSOC models (OVCAR-3, Caov-3), whereas no meaningful decrease was observed in JHOC-8 and only a very small but significant reduction with one shRNA in TOV-21G (Fig. 2*B*). Under low-attachment conditions, *HTRA3* depletion produced a very modest increase in anoikis over time; the effect was more apparent in OCCC models (significant in both cell lines) and smaller in HGSOC (significant in Caov-3, trend in OVCAR-3) (Fig. 2*C*). Together, these data suggest that *HTRA3* supports proliferative capacity in HGSOC, while it may also modestly support resistance to anoikis, particularly in OCCC.

### PRSS23 knockdown reduces proliferation and enhances anoikis sensitivity across ovarian cancer subtypes

**Figure 3:**
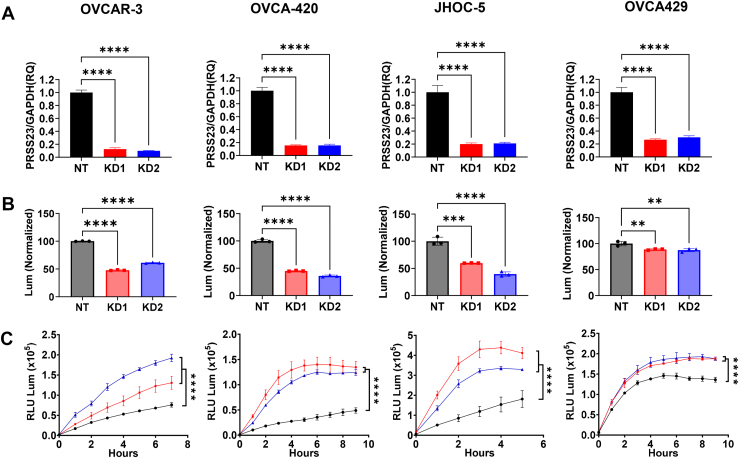
***PRSS23* knockdown reduces proliferation and enhances anoikis sensitivity across ovarian cancer subtypes.***A*, validation of *PRSS23* transcript knockdown in ovarian cancer cell lines following lentiviral transduction with two independent shRNAs (KD1, *red*; KD2, *blue*) and compared to nontargeting control (NT, *black*). Cell lines shown: OVCAR-3 and OVCA-420 (HGSOC) and JHOC-5 and OVCA429 (OCCC). Expression levels were normalized to *GAPDH* and analyzed by qRT-PCR; plots show mean ± SEM. N = 3 per condition. *B*, quantification of proliferation using luminescence-based CellTiter-Glo assays. Data represent percentage proliferation over 16 h relative to NT. N = 3 per condition. *C*, detachment-induced apoptosis under low attachment conditions measured over time using the RealTime-Glo Annexin V apoptosis assay; increased luminescence reflects increased Annexin V signal (phosphatidylserine exposure), consistent with anoikis. N = 3 per condition. Data are presented as mean ± SD with individual data points superimposed. Statistical analysis was performed using one-way ANOVA with Dunnett’s multiple comparisons test for (*A* and *B*) and two-way ANOVA with Dunnett’s multiple comparisons test for (*C*); ∗∗*p*< 0.01, ∗∗∗*p*< 0.001, ∗∗∗∗*p*< 0.0001. HGSOC, high-grade serous ovarian carcinoma; OCCC, ovarian clear cell carcinoma.

We next evaluated whether *PRSS23* contributes to proliferation and detachment survival as hallmarks of metastatic progression. Two independent shRNAs were used to deplete *PRSS23* in OVCAR-3 and OVCA-420 (HGSOC) and JHOC-5 and OVCA429 (OCCC), with qRT-PCR confirming efficient silencing in all four models (Fig. 3*A*). *PRSS23* loss markedly reduced proliferation in OVCAR-3, OVCA-420, and JHOC-5, with a smaller but still significant decrease in OVCA429 (Fig. 3*B*). Under low-attachment conditions, *PRSS23* knockdown enhanced anoikis sensitivity across all cell lines, with large effect sizes in OVCAR-3, OVCA-420, and JHOC-5, and more modest but consistent enhancement in OVCA429 (Fig. 3*C*). Taken together, these data indicate that across histotypes, *PRSS23* promotes both proliferative capacity and resistance to detachment-induced cell death, two prerequisites for peritoneal dissemination. Thus, *PRSS23* emerges as a likely mediator of ovarian cancer metastasis within the peritoneal cavity. Given the larger and more consistent effects of *PRSS23* across both HGSOC and OCCC models, compared with the more modest, histotype-dependent effects observed for *HTRA3*, we next prioritized *PRSS23* for *in vivo* and mechanistic follow-up.

### PRSS23 knockdown limits peritoneal tumor establishment, dissemination, and ascites in intraperitoneal xenografts

**Figure 4:**
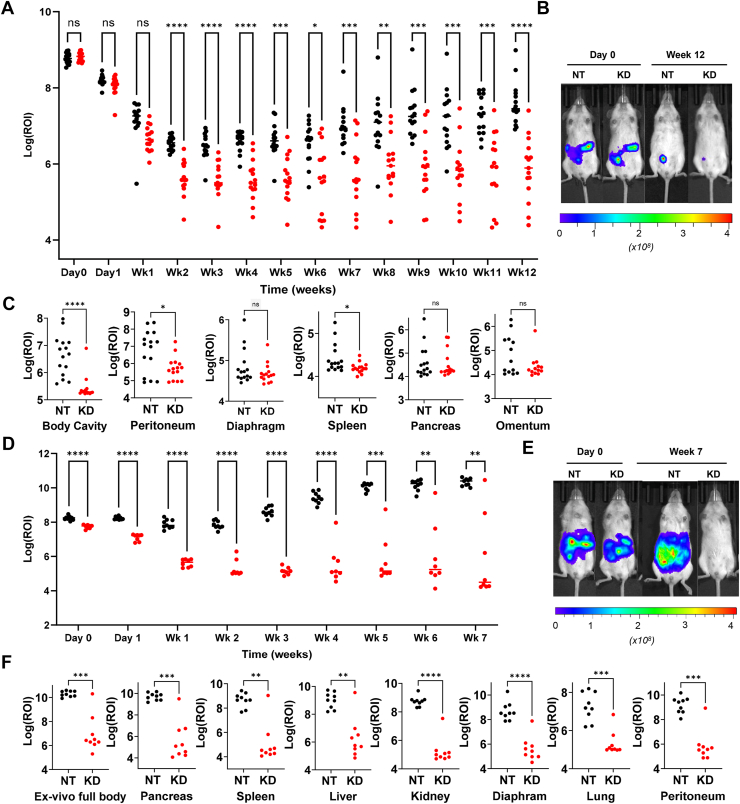
***PRSS23* knockdown limits intraperitoneal tumor burden and dissemination in HGSOC and OCCC xenografts.** Luciferase-expressing ovarian cancer cells transduced with lentiviral constructs encoding nontargeting control (NT, *black*) or *PRSS23* knockdown (KD, *red*) shRNAs were injected intraperitoneally and monitored by IVIS bioluminescence imaging. *A*, OVCA-420 (HGSOC) longitudinal bioluminescence signal plotted as log_10_(ROI) over time (each dot represents an individual animal at that time point; n = 15 per group). *B*, representative IVIS images at day 0 and week 12. *C*, *ex vivo* bioluminescence quantification at OVCA-420 endpoint (12 weeks) in the indicated sites: body cavity, peritoneum, diaphragm, spleen, pancreas, and omentum (each dot represents one animal). *D*, JHOC-5 (OCCC) longitudinal bioluminescence signal plotted as log_10_(ROI) over time (each dot represents an individual animal at that time point; NT, n = 9/KD n = 8). *E*, representative IVIS images are shown at day 0 and week 7. *F*, *ex vivo* bioluminescence quantification of metastatic burden at JHOC-5 endpoint (week 7) in the indicated sites: full body, pancreas, spleen, liver, kidney, diaphragm, lung, and peritoneum (each dot represents one animal). Longitudinal *in vivo* bioluminescence (log_10_-transformed ROI) was analyzed by two-way repeated-measures ANOVA with Geisser–Greenhouse correction, followed by Šídák’s multiple comparisons test at each time point. *Asterisks* above time points indicate Šídák-adjusted *p* values. *Ex vivo* endpoint log_10_(ROI) values were compared between NT and KD groups using a Mann Whitney test. ∗*p*< 0.05, ∗∗*p*< 0.01, ∗∗∗*p*< 0.001, ∗∗∗∗*p*< 0.0001; ns, not significant. HGSOC, high-grade serous ovarian carcinoma; OCCC, ovarian clear cell carcinoma.

To test whether *PRSS23* supports the *in vivo* steps of peritoneal metastasis—survival in suspension, seeding of mesothelial surfaces, and proliferative outgrowth—we used an intraperitoneal xenograft model. Ovarian cancer cells were first transduced with either nontargeting control (NT) or *PRSS23* shRNA (KD), briefly selected with puromycin, and then transduced with a luciferase vector to enable longitudinal imaging. For each model, equal numbers of viable NT and *PRSS23*-KD cells were injected intraperitoneally (OVCA-420: 5 × 10^6^cells/mouse; JHOC-5: 5 × 10^5^cells/mouse), and tumor burden was monitored weekly by bioluminescence imaging. At the study endpoint, *ex vivo* imaging at necropsy was used to quantify dissemination to peritoneal and visceral sites (Fig. 4).

In the OVCA-420 model, both cohorts showed an initial drop in luminescence over the first 1 to 2 weeks, consistent with loss of unattached cells to anoikis, but the decline was greater with *PRSS23* knockdown, and from week 2 onward bioluminescence remained significantly lower at all measured time points through week 12 (Fig. 4, *A* and *B*). Longitudinal analysis confirmed an overall reduction in intraperitoneal tumor burden with *PRSS23* knockdown (repeated-measures ANOVA on log-transformed ROI, *p*< 0.0001). At necropsy, *ex vivo* imaging showed lower signal across several peritoneal sites in KD mice, with significant differences in a subset of organs (Fig. 4*C*), indicating diminished seeding and outgrowth. Together, these data suggest that *PRSS23* supports both early intraperitoneal survival of unattached tumor cells and subsequent establishment of peritoneal lesions in this HGSOC model.

In the JHOC-5 model, NT cells expanded rapidly in the peritoneal cavity, whereas *PRSS23* knockdown produced a progressive decline in bioluminescence over several weeks followed by a low-level plateau, with KD values significantly below controls at all time points through week 7 (Fig. 4, *D* and *E*). Here as well, longitudinal analysis confirmed an overall reduction in intraperitoneal tumor burden with *PRSS23* knockdown (repeated-measures ANOVA on log-transformed ROI, *p*< 0.0001). At necropsy, we harvested organs and performed *ex vivo* imaging, which demonstrated uniformly lower metastatic burden across all examined sites in KD animals (Fig. 4*F*). Notably, ascites was present in every NT mouse in this model (range 88–2112 μl) and absent in all KD mice, consistent with markedly reduced intraperitoneal disease burden when *PRSS23* is silenced. Together, these data indicate that *PRSS23* is required for both early intraperitoneal survival of detached tumor cells and sustained expansion of metastatic disease in this OCCC model.

Across both xenograft models, *PRSS23* knockdown impaired the key steps of peritoneal dissemination—survival in suspension, seeding, and outgrowth—resulting in persistently lower longitudinal bioluminescence and diminished organ involvement. In JHOC-5, this extended to the complete absence of ascites in the KD cohort. Ascites was not observed in either cohort in the OVCA-420 model, consistent with its lower overall tumor burden. Our *in vivo* findings, together with the cell-autonomous roles in anoikis resistance and proliferation observed *in vitro*, identify *PRSS23* as a determinant of intraperitoneal metastatic competence in ovarian cancer.

### PRSS23 knockdown reprograms inflammatory, adhesion, and cell-cycle transcriptional networks in ovarian cancer cells

**Figure 5:**
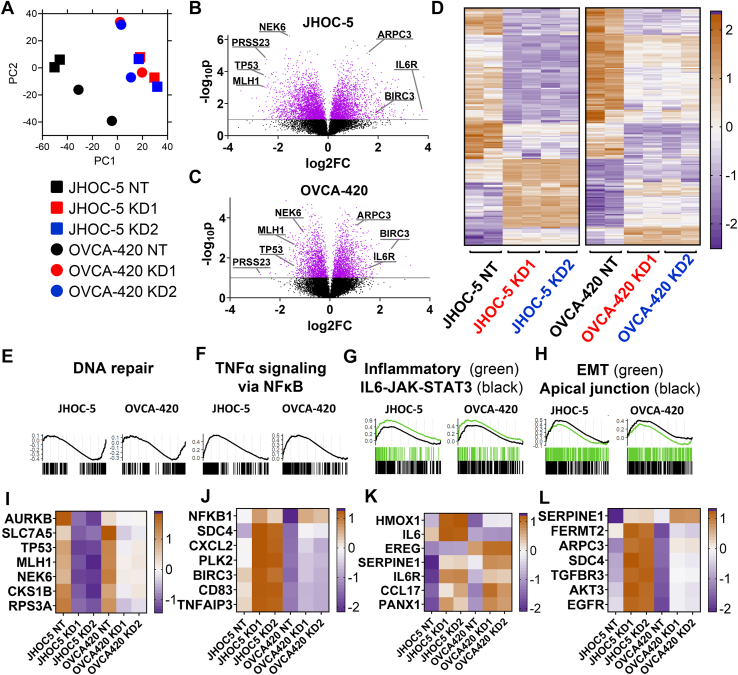
***PRSS23* knockdown induces a conserved transcriptional program of reduced cell-cycle/DNA repair and increased inflammatory and adhesion pathways.** Bulk RNA-seq was performed in JHOC-5 (OCCC) and OVCA-420 (HGSOC) cells transduced with nontargeting control (NT) or two independent *PRSS23* shRNAs (KD1, KD2), N = 2 biological replicates per condition. *A*, cell line–adjusted principal component analysis shows segregation of *PRSS23* knockdown samples from matched NT controls in both models. *B* and *C*, volcano plots for differential expression in JHOC-5 (*B*) and OVCA-420 (*C*) comparing KD *versus* NT. *Horizontal line* marks *p*= 0.01; *purple dots* indicate genes with *p*< 0.01. Selected genes are labeled, highlighting *PRSS23* itself and representative regulators of proliferation and DNA damage response (*NEK6*, *MLH1*, *TP53*), inflammatory signaling (*IL6R*), adhesion/EMT (*ARPC3*) and TNFα signaling (*BIRC3*). *D*, heat map of the core *PRSS23* knockdown signature (genes with |log_2_FC| ≥ 1 in both cell lines with concordant direction), showing clustering of knockdown samples across histotypes. *Brown* indicates higher and *purple* lower expression relative to the gene mean. *E–H*, gene set enrichment analysis (GSEA) of selected MSigDB Hallmark pathways, showing consistent pathway-level changes with *PRSS23* knockdown, including negative enrichment of DNA repair (*E*) and positive enrichment of TNFα signaling *via* NFκB (*F*), inflammatory response (*green*)/IL6-JAK-STAT3 signaling (*black*) (*G*), and epithelial-mesenchymal transition (EMT, *green*)/apical junction (*black*) (*H*), plotted for JHOC-5 and OVCA-420. Barcode plots beneath each curve indicate the positions of Hallmark genes in the ranked expression list. *I–L*, mini-heatmaps illustrating curated gene modules derived from Hallmark gene sets and the core *PRSS23* signature. *I*, G2M checkpoint/DNA repair module. *J*, TNFα/NFκB module. (*K*) IL6/inflammatory module (*HMOX1*, *IL6*, *EREG*, *SERPINE1*, *IL6R*, *CCL17*, *PANX1*) capturing IL6-JAK-STAT3 and inflammatory response module. *L*, EMT/adhesion module. For each module, heatmaps display gene-wise z-scored mean log_2_CPM per condition (JHOC-5 NT, JHOC-5 KD1, JHOC-5 KD2, OVCA-420 NT, OVCA-420 KD1, OVCA-420 KD2; n = 2 RNA-seq replicates averaged). HGSOC, high-grade serous ovarian carcinoma; OCCC, ovarian clear cell carcinoma.

To gain insight into the cell-intrinsic programs through which *PRSS23* supports proliferation, anoikis resistance, and peritoneal spread, we next profiled transcriptional changes following *PRSS23* knockdown in the JHOC-5 and OVCA-420 models used for our *in vivo* studies. We performed bulk RNA-seq on JHOC-5 and OVCA-420 cells transduced with NT or two independent *PRSS23* shRNAs (KD1, KD2). After normalization to log_2_ counts per million (log_2_CPM) and restriction to protein-coding genes, we first examined global sample relationships. A cell line-adjusted principal component analysis (PCA), in which gene-wise means were subtracted within each line to remove baseline JHOC-5–OVCA-420 differences, revealed that *PRSS23*-depleted samples from both models segregated from their matched NT controls along the first two principal components (Fig. 5*A*). Replicates clustered tightly by condition and shRNA, indicating a robust and reproducible knockdown-associated expression shift that was shared across histologic subtypes.

We next performed differential expression analysis comparing KD (KD1 and KD2 combined) *versus* NT within each cell line. Volcano plots showed that *PRSS23* knockdown produced widespread transcriptional changes in JHOC-5 (Fig. 5*B*). OVCA-420 showed a qualitatively similar but quantitatively more modest response, with many genes displaying concordant log_2_FC trends (Fig. 5*C*). In both lines, *PRSS23* itself was among the most strongly downregulated transcripts, validating the efficacy of the shRNAs. Consistent with our functional data, key markers of cell proliferation (*MLH1*, *TP53*, *NEK6*) were commonly downregulated; we also found that markers of altered cell adhesion (*ARPC3*), inflammation (*IL6R*), and tumor necrosis factor-α (TNFα) signaling (*BIRC3*) were upregulated.

To focus on transcriptional alterations shared between models, we defined a “core” *PRSS23* knockdown signature as protein-coding genes with |log_2_FC| ≥ 1 in both JHOC-5 and OVCA-420 and the same direction of change. Heat-map visualization of this 193-gene set, using gene-wise z-scored log_2_CPM across all 12 RNA-seq samples, showed a strikingly coherent pattern: JHOC-5 and OVCA-420 knockdown samples clustered together and were clearly separated from their respective NT controls (Fig. 5*D*). This shared signature contained both downregulated genes enriched for DNA replication, mitotic progression and DNA repair, and upregulated genes associated with inflammatory signaling, extracellular matrix, and cell–cell adhesion.

To interpret these patterns in the context of canonical pathways, we performed gene set enrichment analysis (GSEA) using the MSigDB Hallmark collection, plotting enrichment curves for JHOC-5 and OVCA-420 side-by-side for each pathway (Fig. 5, *E*–*H*). *PRSS23* knockdown led to negative enrichment of the DNA repair Hallmark in both cell lines (JHOC-5 NES = −1.46, FDR = 0.029; OVCA-420 NES = −1.25, FDR = 0.11; Fig. 5*E*), consistent with depletion of repair-associated genes in the core signature. In contrast, TNFα signaling *via* nuclear factor κB (NFκB) was strongly positively enriched in both models (JHOC-5 NES = 2.15, FDR < 2.2 × 10^−16^; OVCA-420 NES = 1.78, FDR = 0.004; Fig. 5*F*). The interleukin-6 (IL6)-Janus kinase (JAK)-signal transducer and activator of transcription 3 (STAT3) and inflammatory response Hallmarks also showed concordant positive enrichment (JHOC-5 NES = 2.06 and 1.52, FDR < 2.2 × 10^−16^ and 0.022; OVCA-420 NES = 1.79 and 1.48, FDR = 0.007 and 0.038, respectively), with closely overlapping enrichment curves in the two cell lines (Fig. 5*G*). Finally, epithelial–mesenchymal transition (EMT) and apical polarity signatures were enriched in both models: EMT (JHOC-5 NES = 1.83, FDR < 2.2 × 10^−16^; OVCA-420 NES = 1.44, FDR = 0.036) together with an apical junction Hallmark in JHOC-5 (NES = 1.48, FDR = 0.026) and the related apical surface Hallmark in OVCA-420 (NES = 1.53, FDR = 0.021; Fig. 5*H*). Collectively, these GSEA results indicate that *PRSS23* knockdown elicits similar patterns of pathway activation and repression in both models, with quantitatively stronger effects in JHOC-5 but clear directional concordance in OVCA-420.

To visualize these pathways at gene-level resolution, we constructed four curated modules by intersecting the significantly enriched Hallmark sets with the core *PRSS23* signature and then plotting gene-wise z-scored mean log_2_CPM values for each condition (Fig. 5, *I*–*L*). The DNA repair module (*AURKB*, *SLC7A5*, *TP53*, *MLH1*, *NEK6*, *CKS1B*, and *RPS3A*) was uniformly reduced in knockdown samples, with a pronounced decrease in JHOC-5 and a more modest but consistent suppression in OVCA-420 (Fig. 5*I*). The TNFα/NFκB module (*BIRC3*, *CD83*, *CXCL2*, *PLK2*, *SDC4*, *NFKB1*, and *TNFAIP3*) showed coordinated upregulation in both lines (Fig. 5*J*). An IL6/inflammatory module (*HMOX1*, *IL6*, *EREG*, *SERPINE1*, *IL6R*, *CCL17*, and *PANX1*) was strongly induced in JHOC-5 and exhibited the same pattern in OVCA-420, although with smaller amplitude (Fig. 5*K*). Finally, the EMT/adhesion module (*SERPINE1*, *FERMT2*, *ARPC3*, *SDC4*, *TGFBR3*, *AKT3*, and *EGFR*) was increased across both models (Fig. 5*L*). These module-level heatmaps support that the pathways highlighted by GSEA are driven by overlapping sets of genes that respond to *PRSS23* loss in a concordant fashion in OCCC and HGSOC cells, reinforcing the existence of a shared *PRSS23*-dependent transcriptional program.

Together, these transcriptomic data demonstrate that *PRSS23* knockdown engages a convergent program of reduced cell-cycle and DNA-repair gene expression coupled with heightened inflammatory, NFκB, IL-6, and adhesion/EMT signaling in both OCCC and HGSOC cells. These pathways provide a mechanistic framework linking *PRSS23* to the proliferation and anoikis phenotypes observed *in vitro* and to the reduced tumor burden and dissemination seen in our xenograft models.

### PRSS23 undergoes glycosylation, prodomain processing, and secretion of the serine protease domain from ovarian cancer cells

**Figure 6:**
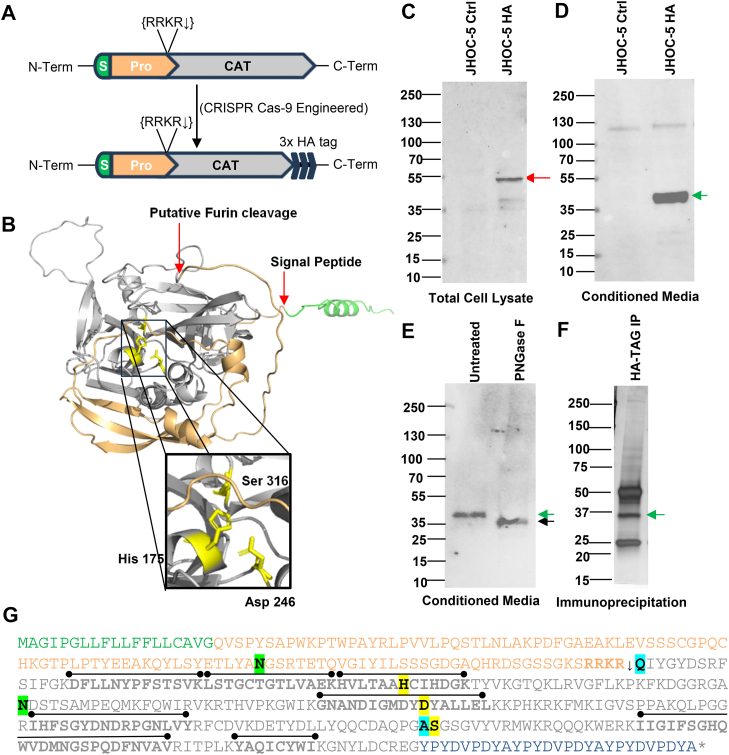
**Endogenous PRSS23 is synthesized as a precursor and secreted as a processed, glycosylated serine protease homology domain.***A*, schematic of predicted PRSS23 domain organization showing the N-terminal signal peptide (S, *green*), prodomain (Pro, *tan*), C-terminal serine protease homology domain (CAT, *gray*), and a potential prodomain processing site/furin recognition motif RRKR. The introduction of a C-terminal 3×HA tag by CRISPR into the endogenous locus is illustrated. *B*, AlphaFold-predicted structure of PRSS23, colored as above, highlights catalytic triad residues His175, Asp246, and Ser316 (*yellow*). Potential processing sites are shown with *red arrows*. *C*, anti-HA immunoblot of whole-cell lysates from JHOC-5 parental (Ctrl) and PRSS23-3×HA knock-in cells (HA) shows a ∼46 kDa precursor species (*red arrow*). *D*, anti-HA immunoblot of conditioned media demonstrates secretion of a ∼36 kDa PRSS23 species (*green arrow*) consistent with a processed protease homology domain fragment. *E*, PNGase F (N-linked glycosidase) digestion of conditioned media shifts the band to ∼32.4 kDa (*black arrow*), matching the predicted molecular weight of the deglycosylated protease homology domain. *F*, anti-HA immunoprecipitation from conditioned medium followed by SDS-PAGE with silver staining shows recovery of the ∼36 kDa secreted PRSS23 species (*green arrow*). In-gel trypsin digestion and LC-MS/MS of this band identified PRSS23 peptides mapping exclusively to the protease homology domain downstream of the RRKR site. *G*, PRSS23 sequence (domains colored as above) showing the RRKR processing site (*bold*), predicted glycosylation sites (*green highlight*), catalytic triad residues (*yellow highlight*), and peptides recovered by LC-MS/MS from the secreted product (*black lines above sequence*).

Having established a role for *PRSS23* in peritoneal dissemination, we next asked how the protein is processed and whether it is secreted by ovarian cancer cells. AlphaFold modeling of PRSS23 predicted a modular architecture with an N-terminal signal peptide, an extended prodomain, and a C-terminal S1 protease domain incorporating a canonical Ser-His-Asp catalytic triad arrangement (His175, Asp246, and Ser316) (Figs. 6, *A* and *B* and S2*A*). Furthermore, to assess whether appending a short C-terminal epitope tag might plausibly perturb the structure, we also generated AlphaFold models of the serine protease domain incorporating either a C-terminal 3 × hemagglutinin (HA) or 6 × His extension. Superposition of tagged and untagged models predicted no appreciable change in the protease domain core or catalytic triad geometry (Fig. S2*B*). To detect endogenous PRSS23 in ovarian cancer cells, we first assessed a variety of commercial antibodies using *PRSS23* knockdown as a specificity control but found none sufficiently specific with good lot-to-lot consistency in our hands. We therefore used CRISPR/Cas9 to introduce a C-terminal 3 × HA tag at the endogenous *PRSS23* locus into both *PRSS23* alleles in JHOC-5 cells (Fig. 6*A*). Sanger sequencing confirmed precise in-frame knock-in (Fig. S3*A*), and anti-HA western blots detected the tagged protein in cell lysates and conditioned media using two independent monoclonal antibodies (Figs. 6, *C* and *D* and S3, *B* and *C*).

Biochemical characterization of HA-tagged PRSS23 showed that it is synthesized as a precursor and secreted as a processed protease domain fragment. In whole-cell lysates, anti-HA western blotting revealed a ∼46 kDa species consistent with the full-length precursor (Fig. 6*C*, *red arrow*). In conditioned media, a dominant ∼36 kDa band (∼10 kDa smaller than the precursor) was detected (Fig. 6*D*, *green arrow*), suggesting secretion of a proteolytically processed PRSS23 species. PNGase F treatment of conditioned media shifted this band to ∼32 kDa (Fig. 6*E*), matching the predicted mass of the unglycosylated protease domain plus 3 × HA tag and indicating N-linked glycosylation of the secreted protease domain fragment, most likely at Asn207 (the only consensus N-glycosylation sequon within the protease domain; Fig. 6*G*). To directly define the secreted form, we immunoprecipitated PRSS23-3×HA from conditioned media, excised the ∼36 kDa band, and identified peptides by LC-MS/MS. All recovered peptides mapped downstream of the canonical RRKR furin recognition sequence and within the protease domain, with no peptides detected in the prodomain (Fig. 6, *F* and *G*). These data indicate that in ovarian cancer cells PRSS23 enters the secretory pathway as a precursor protein that is processed, likely at the canonical RRKR motif, and secreted as a glycosylated protease domain fragment after removal of the prodomain.

### Recombinant PRSS23 protease domain shows no Ser316-dependent peptidase activity and lacks the canonical S1 family activation switch

Because PRSS23 is secreted from ovarian cancer cells as a processed serine protease domain fragment, we next asked whether the PRSS23 protease homology domain exhibits detectable proteolytic activity on peptide substrates. To enable biochemical screening, we pursued recombinant expression of PRSS23, including full-length and protease domain constructs across mammalian, yeast, and bacterial systems. While most approaches yielded only trace recoverable material, we obtained limited soluble expression of the PRSS23 protease domain in bacteria as a SUMO fusion (Fig. S4*A*). SUMO protease cleavage released the PRSS23 protease domain (Fig. S4*B*). Because yields were insufficient to allow purification of the released protease domain to homogeneity, peptide substrate assays were performed using the postcleavage reaction mixtures after normalizing the amount of released PRSS23 protease domain in each mixture by densitometry of the released PRSS23 band.

**Figure 7:**
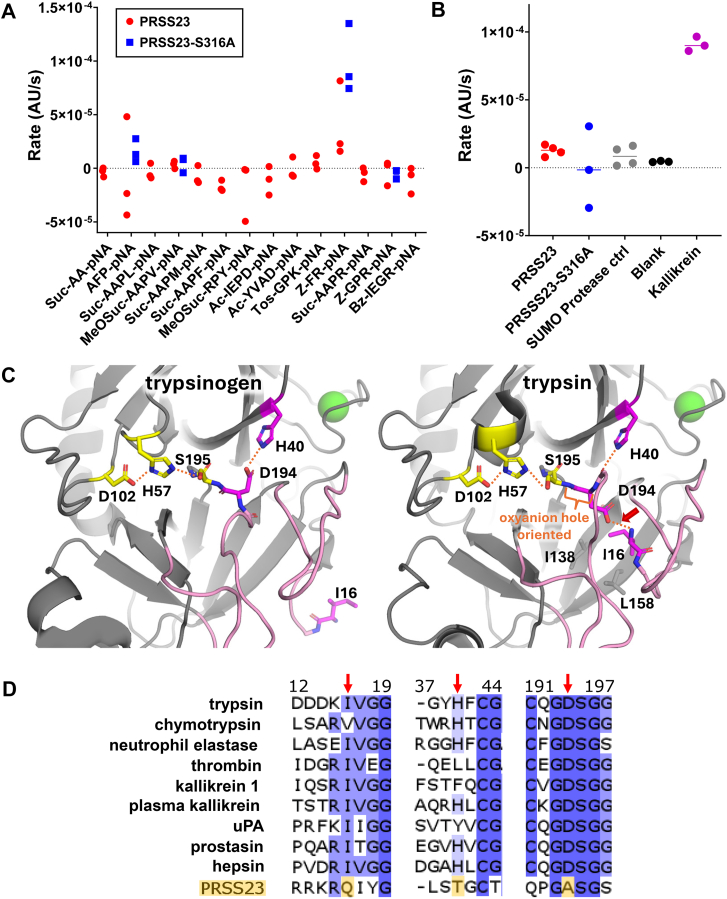
**Recombinant PRSS23 protease domain shows no Ser316-dependent peptidase activity and lacks the canonical Ile16-Asp194 activation switch.***A*, WT PRSS23 protease domain (300 nM) was assayed against a panel of chromogenic peptide-pNA substrates (200 μM each; substrates indicated). PRSS23-S316A (300 nM) was assayed in parallel for a subset of substrates, including those for which WT PRSS23 exhibited apparent rates above baseline. N = 3 per condition. *B*, confirmatory assays on substrate Z-FR-pNA (200 μM) using an independently produced pair of WT and PRSS23-S316A protein preparations (300 nM each), with matched control reactions containing SUMO protease alone (“SUMO protease ctrl”), buffer alone (“Blank”), and kallikrein (2 nM) as a positive control. *Panels A* and *B* used independent protein preparations. Rates are plotted as reaction slopes (AU/s); each point represents an independent assay replicate (N = 3–4 as shown; *dotted line* indicates zero). *C*, structural comparison of trypsinogen (PDB: 2TGT (71)) and trypsin (PDB: 2RA3 (72)) illustrating the Ile16-Asp194 activation switch (chymotrypsin numbering). Catalytic triad residues Asp102, His57, and Ser195 are shown as *yellow sticks*, and activation switch residues Ile16, Asp194, and His40 are shown as *magenta sticks*. In the zymogen (left), Asp194 forms an alternative H-bond with His40, activation domain loops (*pink*) are in a partially disordered, substrate-incompatible state, and the oxyanion hole is not properly oriented. In the active protease (*right*), cleavage generates a new Ile16 N terminus that inserts into the Ile16 pocket and forms the characteristic Ile16-Asp194 salt bridge (*red arrow*), stabilizing activation domain loops in a substrate-compatible conformation and orienting the oxyanion hole. Structure images were rendered in PyMOL. *D*, multiple sequence alignment of representative human S1 serine proteases *versus* PRSS23, highlighting residues that comprise the activation switch (*red arrows*). PRSS23 substitutes Gln at the predicted mature N terminus and Ala at the Asp194-equivalent position, eliminating the canonical Ile16-Asp194 salt bridge; the His40-equivalent residue is also substituted. Alignment was created in Clustal Omega and visualized in JalView colored by BLOSUM62 conservation score. Sequences were obtained from UniProt: trypsin P07477; chymotrypsin Q99895; neutrophil elastase P08246; thrombin P00734; kallikrein 1 P06870; plasma kallikrein P03952; uPA P00749; prostasin Q16651; hepsin P05981; PRSS23 O95084.

To broadly survey potential PRSS23 peptidase activity across the range of P1 specificities described for S1 serine proteases, we generated a matched Ser316Ala variant of the recombinant protease domain and assayed wildtype (WT) and Ser316Ala preparations in parallel across a panel of chromogenic p-nitroanilide (pNA) peptide substrates spanning diverse P1 residues (Fig. 7*A*). This assay design also allowed us to rigorously evaluate a preliminary observation of Z-FR-pNA hydrolysis by recombinant PRSS23 that we previously reported in a published abstract (31), prior to availability of the catalytic serine mutant control. Across this substrate panel, we did not observe reproducible substrate turnover above background that depended on the putative catalytic serine; WT and Ser316Ala preparations exhibited similar rates for all substrates tested (Fig. 7*A*). Z-FR-pNA showed the highest apparent rates in this screen, but the Z-FR-pNA signal was not reduced by Ser316 substitution (Fig. 7*A*). We next repeated the Z-FR-pNA assay using an independently produced pair of WT and Ser316Ala preparations, assayed under the same conditions as the initial screen, and included matched control reactions containing SUMO protease alone, buffer alone, and kallikrein as a positive control (Fig. 7*B*). In this follow-up, the WT and Ser316Ala preparations again exhibited similar Z-FR-pNA turnover rates, but the absolute rates were substantially lower than in the initial preparation (Fig. 7*B*). The variability in low-level Z-FR-pNA turnover between independent preparations, coupled with the absence of Ser316 dependence, is most consistent with a variable copurifying background activity rather than intrinsic PRSS23 catalysis. Together, these controlled peptide substrate assays did not reveal Ser316-dependent proteolytic activity of recombinant PRSS23 across the broad substrate panel.

Given the intact catalytic triad (Fig. 6*B*), we next examined other sequence features of PRSS23 that might explain the absence of Ser316-dependent peptidase activity. In classical trypsin-like serine proteases, removal of the prodomain (or a shorter propeptide) is coupled to a conserved Ile16-Asp194 activation switch that assembles the active site (Fig. 7*C*) (32, 33, 34). The activating cleavage exposes an Ile or Val at position 16 (chymotrypsin numbering), and its side chain inserts into a hydrophobic “Ile16 pocket,” positioning the free α-amino group to form a buried salt bridge with Asp194 (Fig. 7*C*). This Ile16-Asp194 activation switch orders the previously flexible activation domain loops, thereby creating the substrate-binding S1 pocket and oxyanion hole required for efficient catalysis. Alignment of PRSS23 with a selection of diverse human S1 proteases shows that despite preserving the canonical Ser-His-Asp catalytic triad (Fig. 6, *B* and *G*), PRSS23 substitutes Gln129 at the predicted mature N terminus (Gln-Ile-Tyr…) where active S1 proteases uniformly harbor Ile/Val, and Ala315 at the Asp194-equivalent position (Figs. 7*D* and S5). PRSS23 also lacks His40 which, in a subset of S1 proteases, coordinates with Asp194 in the zymogen state and can help stabilize the inactive conformation prior to activation (35, 36); in PRSS23, the equivalent residue is Leu250. Notably, these activation switch substitutions are conserved across vertebrate PRSS23 orthologs (Fig. S6, *A*–*F*). The His-Asp-Ser catalytic triad positions are likewise conserved across PRSS23 orthologs (Fig. S6, *A*–*F*). Phylogenetic analysis identifies PRSS35 as the only close vertebrate paralog of PRSS23 (Fig. S7). Like PRSS23, PRSS35 lacks the canonical Ile16-Asp194 activation switch and, in most species, substitutes Thr for the catalytic Ser (Fig. S6, *A*–*F*), a substitution not expected to support catalytic activity (37), consistent with its annotation as a noncatalytic peptidase homolog. In aggregate, the conserved substitutions remove all components of the Ile16-Asp194 activation switch, suggesting that PRSS23 may be unable to stabilize a classical active conformation even after prodomain removal and instead may retain a zymogen-like or alternative state despite maturation. Together, these observations provide a plausible structural basis for the lack of detectable activity of the recombinant PRSS23 preparations on peptide substrates.

### PRSS23 promotes ovarian cancer cell anoikis resistance and proliferation through a protease-independent mechanism

Given that recombinant PRSS23 preparations did not reveal Ser316-dependent peptidase activity and that PRSS23 lacks the canonical Ile16-Asp194 activation switch, we next tested whether it behaves as an active serine hydrolase in ovarian cancer cells and whether the “catalytic” serine Ser316 is required for its phenotypic functions. First, we generated JHOC-5 cells in which the endogenous 3×HA-tagged *PRSS23* locus was further edited by CRISPR/Cas9 to introduce a Ser316Ala substitution at the site of the putative catalytic serine into both alleles; Sanger sequencing confirmed precise all-allelic editing at the Ser316 codon (Fig. S3*D*).

**Figure 8:**
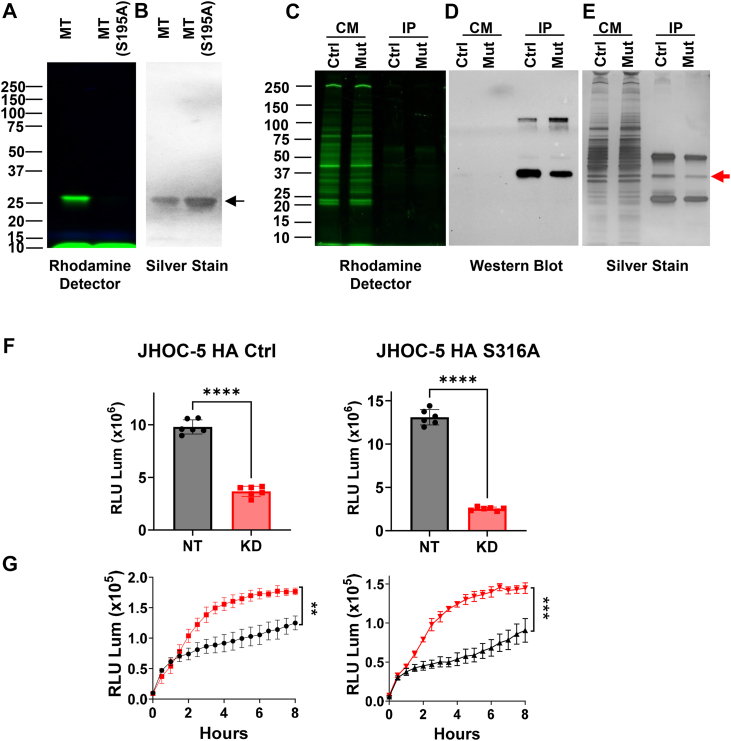
**Secreted PRSS23 lacks detectable serine hydrolase activity, and PRSS23-dependent phenotypes do not require the putative catalytic serine.***A* and *B*, validation of activity-based serine hydrolase probe workflow using recombinant mesotrypsin (MT) and a catalytic-null S195A mutant labeled with TAMRA-FP; probe labeling was detected by in-gel rhodamine fluorescence (*A*), with protein loading shown by silver stain (*B*). *C*, concentrated conditioned media from JHOC-5 PRSS23-3×HA (Ctrl) and JHOC-5 PRSS23-3×HA S316A (Mut) cells was labeled with TAMRA-FP. Total labeled conditioned media (CM) and anti-HA immunoprecipitates (IP) were analyzed by in-gel rhodamine fluorescence. Multiple probe-reactive secreted hydrolases are detected in CM; however, no probe-reactive band corresponding to PRSS23 is detected at the expected molecular weight in either CM or anti-HA IP fractions. *D*, anti-HA immunoblot confirms efficient immunoprecipitation and enrichment of secreted PRSS23-3×HA from labeled conditioned media. *E*, silver stain of the anti-HA immunoprecipitates confirms recovery and enrichment of the processed PRSS23 species (*red arrow*). Prominent bands at ∼50 and ∼25 kDa correspond to IgG heavy and light chains from the immunoprecipitating antibody. Despite robust enrichment, rhodamine detection failed to detect probe labeling of PRSS23 in (*C*), indicating minimal or absent catalytic activity. *F*, proliferation with shRNA-mediated PRSS23 depletion in WT PRSS23-3×HA *versus* S316A PRSS23-3×HA backgrounds reveals a similar dependency on PRSS23 irrespective of mutational status. WT and S316A lines were assayed side-by-side in the same experiment; data are plotted as raw luminescence (not normalized) to permit direct comparison between genotypes. N = 6 per condition. *G*, detachment-induced apoptosis under low-attachment conditions after *PRSS23* depletion in WT *versus* S316A backgrounds measured by the RealTime-Glo Annexin V apoptosis assay reveals similar sensitization to anoikis upon knockdown, irrespective of mutation status. WT and S316A lines were assayed side-by-side in the same experiment; data are plotted as raw luminescence (not normalized) to permit direct comparison between genotypes. N = 6 per condition. Together, these data indicate that PRSS23-dependent proliferation and anoikis resistance phenotypes are independent of the putative catalytic serine.

Next, we used an ActivX TAMRA-FP serine hydrolase probe, a fluorophosphonate activity-based probe that covalently labels the catalytic serine of active serine hydrolases and has been widely validated as a broad, sensitive reporter of serine hydrolase activity across diverse enzyme families (38). As a positive control, we labeled recombinant human mesotrypsin and its catalytic-null mutant mesotrypsin-S195A. The probe robustly labeled WT mesotrypsin but not the S195A mutant (Fig. 8, *A* and *B*), confirming that the probe and detection workflow report *bona fide* serine protease activity.

We then applied the probe to conditioned media from JHOC-5 cells expressing HA-tagged WT PRSS23 or the S316A mutant. Labeling of equivalent volumes of conditioned media from WT and S316A lines yielded a rich pattern of TAMRA-FP-reactive secreted hydrolases over a broad molecular weight range (Fig. 8*C*), consistent with the validated probe reactivity in control experiments (Fig. 8, *A* and *B*). However, no fluorescent band was uniquely present in the WT sample or absent in the S316A mutant at the expected ∼36 kDa size, as would be expected if PRSS23 were labeled. To test directly whether PRSS23 itself was labeled, we immunoprecipitated PRSS23-3×HA from the labeled media. Western blotting and silver stain confirmed strong enrichment of the processed PRSS23 protease domain from both WT and S316A cultures (Fig. 8, *D* and *E*, *red arrow*), yet no corresponding TAMRA-FP signal was detectable at that molecular weight in the immunoprecipitates in either genotype (Fig. 8*C*), despite robust labeling of multiple other secreted serine hydrolases in the same conditioned media samples. Across multiple probe concentrations, incubation times, and exposures in repeated experiments, we never observed a discrete probe-reactive band that could be assigned to PRSS23, for either the WT or S316A variant.

Given this lack of detectable serine hydrolase activity, we next asked whether the putative catalytic serine is required for PRSS23-dependent cellular phenotypes. In JHOC-5 cells expressing either HA-tagged WT or S316A PRSS23, shRNA-mediated knockdown reduced proliferation to a similar extent and comparably enhanced anoikis sensitivity under low-attachment conditions (Fig. 8, *F* and *G*). Thus, loss of Ser316 does not measurably alter the cellular responses to PRSS23 depletion. Taken together with the disrupted Ile16-Asp194 activation switch inferred from sequence analysis, these findings indicate that secreted PRSS23 lacks detectable serine protease activity. Its ability to promote ovarian cancer cell anoikis resistance and proliferation is therefore independent of its putative catalytic serine, consistent with a protease-independent mechanism of action.

## Discussion

We began with a clinical outcomes-based survey of the S1 serine protease family in ovarian cancer, in which *HTRA3*, *TMPRSS12*, and *PRSS23* genes showed the strongest associations with poor OS, although *TMPRSS12* transcripts were not detectable in our cell line panel. Subsequent functional interrogation revealed modest, histotype-dependent effects for *HTRA3* but consistently implicated *PRSS23* as a driver of proliferation, anoikis resistance, and intraperitoneal dissemination across ovarian cancer histotypes. RNA-seq after *PRSS23* knockdown revealed a shared transcriptional program linking *PRSS23* to proliferative and detachment-survival circuits across histotypes. In parallel, biochemical and sequence analyses showed that PRSS23 is secreted as a processed protease domain fragment yet lacks the canonical Ile16-Asp194 activation switch, shows no detectable reactivity with an activity-based serine hydrolase probe or with a panel of peptide substrates, and retains function when the putative catalytic serine is mutated. Together, these data support the hypothesis that PRSS23 functions as a secreted serine pseudoprotease whose prometastatic effects in ovarian cancer are mediated by nonproteolytic mechanisms.

Catalytically inactive enzyme homologs, or pseudoenzymes, are now recognized as a substantial and functionally diverse group rather than rare curiosities. Within the S1 protease family, multiple serine protease homologs have lost measurable proteolytic activity yet remain essential for developmental, immune, and hemostatic processes, while still adopting the canonical chymotrypsin-like fold (39, 40). Although mutations within the catalytic Ser-His-Asp triad are the most obvious route to serine pseudoprotease evolution, work in other systems demonstrates that the triad is necessary but not sufficient for proteolysis. Catalytic efficiency depends on precise positioning of enzyme catalytic residues and substrates within a properly formed active site, a principle recognized in early “orbital steering” models of catalysis (41, 42). Structural studies of several serine pseudoproteases have shown that activity can be abolished not only by changes in catalytic residues themselves but also by distortions of the surrounding active site architecture that interfere with these positioning effects. Scabies mite inactivated protease paralogs are S1-family proteins that retain the chymotrypsin-like fold but harbor substitutions and loop rearrangements in and around the catalytic cleft (43). Residues that normally form the oxyanion hole and substrate specificity pocket are displaced, mispositioning the serine nucleophile and yielding a geometry incompatible with catalysis (43). Similarly, CspC and CspA, members of the subtilisin-like (S8) clostridial Csp family in *Clostridioides difficile* and related Peptostreptococcaceae, carry an intact or partial catalytic triad yet lack activity due to rearranged active site loops and occlusion of substrate binding (44). Notably, restoring missing catalytic triad residues in scabies mite inactivated protease paralog and Csp family members fails to restore proteolytic function (43, 44), further underscoring the importance of active site architecture for catalysis. Thus, sequence-based annotation focused solely on the catalytic triad will tend to overestimate catalytic competence and underestimate the true prevalence of serine pseudoproteases, as appears to be the case for PRSS23.

PRSS23 provides a distinct example, in which the catalytic triad is preserved, but the conformational switch to activate its zymogen is dismantled. In classical S1 proteases, the zymogen exists as a conformational ensemble in which four activation domain loops are disordered, the substrate-binding specificity pocket is incompletely formed, and the oxyanion hole is not preorganized (32, 33). The activating cleavage exposes an Ile (or Val) at the new N terminus; its side chain tucks into a hydrophobic pocket and positions the free α-amine to form a buried salt bridge with Asp194, bringing order to the active site (32). Mutational and thermodynamic analyses of trypsinogen activation show that hydrophobic packing of Ile16 contributes ∼4 to 5 kcal/mol and the Ile16-Asp194 salt bridge a further ∼2 to 3 kcal/mol of stabilization to the active conformation, together shifting the conformational equilibrium by ∼10^6^- to 10^7^-fold toward the catalytically competent state (34). Consistent with the centrality of this switch, zymogen-state trypsinogen is even less catalytically active than catalytic triad mutants of trypsin (36, 45); thus, failure to order the activation domain that is critical for substrate binding and transition state stabilization can “break” a serine protease even more profoundly than loss of the nucleophile and acid/base. While the vast majority of S1 proteases rely on this newly generated N-terminal Ile/Val-Asp194 ion pair to stabilize the active conformation, the few well-characterized deviations from N-terminal insertion still depend on an acidic residue at the 194 position. For example, the X-ray structure of single-chain tissue-type plasminogen activator shows that Lys156 can insert into the activation pocket and form a Lys156-Asp194 salt bridge prior to activation cleavage (46). Likewise, in complement C2a, the Asp194-equivalent residue is Glu and forms an alternative Glu194-Arg salt bridge that partially substitutes for the canonical N-terminal ion pair (47). By contrast, PRSS23 substitutes Ala at the Asp194-equivalent position, eliminating the acidic anchor used in both canonical and described noncanonical stabilization mechanisms. Thus, despite preserving the Ser-His-Asp catalytic triad, complete loss of the activation switch in PRSS23 might be expected to prevent stabilization of a catalytically productive conformation, instead biasing toward a zymogen-like state or alternative, likely noncatalytic, conformation.

These observations raise the question of how PRSS23, as a secreted serine pseudoprotease, promotes anoikis resistance and proliferative outgrowth. Functionally, serine pseudoproteases can be grouped by the partners they bind; common mechanisms include serving as receptor ligands, noncatalytic cofactors/regulatory subunits in protease complexes, or scaffolds/organizers of other protein assemblies (39, 40). Ligand pseudoproteases, such as hepatocyte growth factor and the related macrophage-stimulating protein, use the S1 protease fold as a receptor binding epitope. Hepatocyte growth factor and macrophage-stimulating protein acquire biological activity upon proteolytic cleavage of their zymogen-like precursors into two-chain forms; the β-chain serine protease homology domain binds the Sema domain of tyrosine kinase receptor MET or RON, respectively, with the pseudoactive site region forming a core recognition interface (48, 49). Other pseudoproteases regulate protease cascades as noncatalytic cofactors or decoys. For example, protein Z binds the protein Z-dependent protease inhibitor on phospholipid surfaces to accelerate inhibition of activated coagulation factor X (50). Another example is the viper venom serine protease homolog VaaSPH-1, which binds activated factor VIII and competitively blocks factor IXa engagement in the intrinsic tenase complex that generates activated coagulation factor X (51). Scaffold-type pseudoenzymes organize higher-order assemblies, a principle well established among pseudokinases (52). Among S1 serine pseudoproteases, a scaffolding mechanism is illustrated by haptoglobin, which uses its inactive serine protease domain to capture free hemoglobin and presents a protruding loop recognized by the scavenger receptor CD163 to promote endocytic clearance (53, 54, 55).

In this framework, the secretion of PRSS23 as a processed protease domain, lacking enzyme activity, suggests a function in ovarian cancer as an extracellular ligand or adaptor/scaffold, potentially engaging an unknown receptor or receptor/adhesion complex. Such a mechanism would be consistent with its autocrine effects on survival and proliferation, as we observe in ovarian cancer cells, and its impact on downstream transcriptional programs. Furthermore, extensive substitutions and insertions/deletions across the PRSS23 protease domain, relative to catalytic family members (Fig. S5), are likely to remodel exposed loops and repurpose its surface for new binding interactions. Conversely, a potential role as a regulator or decoy within a defined protease cascade seems less likely because PRSS23 lacks close homology to any S1 proteases annotated as catalytically active enzymes. Its nearest relatives among characterized enzymes in a search for structural homologs possess only 20 to 23% sequence identity with PRSS23 across the serine protease domain.

Consistent with a role for PRSS23 in outside-in signaling, our RNA-seq analyses suggest that *PRSS23* sits at the nexus of proliferative, stress/inflammatory, and adhesion programs shared between ovarian clear cell and high-grade serous cancers. *PRSS23* knockdown produced a coherent transcriptional response in both JHOC-5 and OVCA-420, despite substantial differences in the baseline transcriptomic profiles of these cell lines; this concordance across models argues that *PRSS23* acts upstream of a convergent axis. Genes involved in DNA replication, mitotic progression, and DNA repair were consistently downregulated, providing a direct rationale for the proliferation defect. Conversely, *PRSS23* depletion induced cytokine and inflammatory signaling modules, including TNFα signaling *via* NFκB and IL6-JAK-STAT3 pathways. EMT, apical junction, and related adhesion gene sets were also enriched, consistent with altered attachment signaling and a compensatory stress response when *PRSS23* is lost. Together, these shared changes provide mechanistic context for reduced proliferation, enhanced anoikis sensitivity, and diminished peritoneal dissemination *in vitro* and in intraperitoneal xenograft models. They also align with the clinical association of high *PRSS23* expression with poor outcome in ovarian cancer cohorts. Together with biochemical evidence that PRSS23 is catalytically inert, this transcriptomic program is consistent with PRSS23 acting as a nonproteolytic soluble effector of outside-in signaling that may buffer tumor cells against detachment-induced death and genotoxic stress. This pseudoprotease view also provides context for revisiting reported PRSS23 functions in normal physiology and other malignancies.

Beyond ovarian cancer, *PRSS23* has been linked to ovarian biology, cardiac morphogenesis, and multiple malignancies, through expression and genetic perturbation studies (29). Although some studies have presumed proteolytic mechanisms based on protein annotation, putative PRSS23 catalytic activity remains unvalidated biochemically. In the mouse ovary, *Prss23* transcripts are expressed in granulosa cells during follicular development (56) and elevated in follicles undergoing atresia (apoptotic follicle degeneration) (57). In zebrafish cardiac valvulogenesis, *prss23* acts upstream to promote Snail-driven endothelial-to-mesenchymal transition (28). In estrogen receptor-positive breast cancer models, PRSS23 is estrogen-responsive and supports proliferative outgrowth (58). In gastric cancer, *PRSS23* has been connected to tumor cell survival and to stromal/immune remodeling, including regulation of fibroblast growth factor 2 and tumor-associated macrophage infiltration (59). Mechanistically, it has been reported to bind eukaryotic initiation factor 4E (eIF4E) *via* the trypsin-like domain to promote gastric tumorigenesis and progression *via* the eIF4E-c-Myc axis (60). Notably, eIF4E binding and progrowth phenotypes were retained in catalytic triad mutants of PRSS23 in that system (60), providing an independent line of evidence for nonproteolytic PRSS23 function. Together with the disrupted activation mechanism and lack of detectable hydrolase activity shown in our present work, these observations support a model in which PRSS23 may act broadly through nonproteolytic, binding-driven mechanisms across disparate contexts.

Collectively, this work identifies PRSS23 as a determinant of peritoneal metastatic competence in both HGSOC and OCCC and demonstrates that its protumorigenic function is protease independent. PRSS23 is secreted as a processed protease-like domain but lacks detectable serine hydrolase activity, and prometastatic phenotypes persist after mutation of the putative catalytic serine, suggesting that PRSS23 may warrant reclassification as a pseudoprotease associated with adverse clinical outcomes. A central next step will be to define the extracellular binding partner(s) and interaction surfaces that connect PRSS23 to outside-in signaling and to the convergent survival, cell-cycle, and attachment programs revealed by its knockdown transcriptome. From a translational perspective, because PRSS23 is secreted and important for intraperitoneal tumor establishment, intercepting its production, secretion, or receptor engagement could be explored to limit peritoneal dissemination. More broadly, these findings argue that protease-centric views of the S1 family should be expanded to include serine pseudoproteases, whose binding functions can occupy critical control points in metastatic progression.

## Experimental procedures

### Kaplan–Meier survival analysis

The data presented in Tables 1, S1 and Fig. 1 were obtained from the Kaplan–Meier plotter database (http://kmplot.com/analysis/) as described by Győrffy, B (61, 62). This analysis focuses on 15 ovarian cancer cohorts available in the NCBI Gene Expression Omnibus (https://www.ncbi.nlm.nih.gov/geo/) and the Genomic Data Commons Data Portal (https://portal.gdc.cancer.gov/). The accession numbers for the cohorts included in the analyses are as follows: GSE14764, GSE15622, GSE18520, GSE19829, GSE23554, GSE26193, GSE26712, GSE27651, GSE30161, GSE3149, GSE51373, GSE63885, GSE65986, GSE9891 and TCGA (OV -ovarian cancer).

The Kaplan–Meier plotter database was interrogated sequentially with a list of genes representing all known and putative S1 family peptidases in the *Homo sapiens* genome, as obtained from the Merops Peptidase Database on August 10th, 2024 (https://www.ebi.ac.uk/merops/) (19). Genes annotated as nonpeptidase homologs were omitted. Genes that were not represented among the datasets included in the Kaplan–Meier plotter database were also omitted. For the analysis of the remaining 93 genes, default settings were used with the following adjustments: for each survival analysis (OS, PFS, and postprogression survival), the "only JetSet best probe set" option was used to select the optimal probe taking into account specificity, coverage, and degradation resistance (63). The Kaplan–Meier plot was generated by clicking "Draw Kaplan–Meier plot." The HRs and *p*-values were manually recorded in a Microsoft Excel sheet. The same Kaplan–Meier plotter settings were applied to a small set of previously reported ovarian cancer prognostic genes for contextual comparison (Table S2).

### Cell lines and culture conditions

JHOC-5, JHOC-7, JHOC-8, and JHOC-9 were purchased from RIKEN BioResource Research Center Japan. IGROV-1, OVISE, and OVTOKO were obtained from BCCRC. OVCA-420, Caov-3, and OVCAR-3 were a gift from Dr John A. Copland’s lab at Mayo Clinic. RMG-1, OVCA429, TYK-nu, KK, Koc7, and OV207 were obtained from Dr Gottfried Konecny at UCLA. HEK 293T cells were a gift from Dr Aubrey Thompson’s lab at Mayo Clinic. All mammalian cultures were propagated from seed stocks and distribution stocks that were validated by short tandem repeat genotyping (PowerPlex 16 HS platform; Promega) and tested for *mycoplasma* contamination (eMYCO PLUS; iNtRON Biotechnologies), through Genetica DNA Laboratories. Cell cultures initiated from short tandem repeat-validated distribution stock aliquots were passaged and used in experiments for up to ∼1 month (∼2 months for slow growing JHOC-8), so long as phenotypic characteristics in culture remained consistent. All cell lines were maintained at 37 °C in a humidified incubator with 5% CO_2_ and 95% humidity. OVCA-420 and Caov-3 cells were cultured in high-glucose Dulbecco's modified Eagle's medium (DMEM) (Cat# 11965) supplemented with sodium pyruvate, 5% fetal bovine serum (FBS), 1× nonessential amino acids, and 1% penicillin-streptomycin-amphotericin B. OVCA429 cells were cultured in MEM Alpha 1× (Corning Cellgro) with 10% FBS. JHOC-5 and JHOC-8 cells were cultured in DMEM/F12 medium containing 10% FBS and 0.1% MEM-nonessential amino acids. TOV-21G cells were grown in a 1:1 mixture of MCDB 105 medium (final sodium bicarbonate concentration: 1.5 g/L) and medium 199 (final sodium bicarbonate concentration: 2.2 g/L). All cultures were passaged at a 1:2 ratio every 3 days or upon reaching 80 to 90% confluency.

### Detection of PRSS23 in cell lysates and conditioned media

Total protein was extracted from cultured cells using RIPA buffer (Cell Signaling, Cat# 9803), followed by brief sonication and centrifugation (15,000 rpm, 10 min, 4 °C). Protein concentration was determined using the Bradford assay (Bio-Rad, Cat# 5000002). For secreted PRSS23 detection, JHOC-5 cells stably expressing PRSS23-3×HA were cultured to 90% confluency, washed extensively with cold PBS, and incubated in serum-free DMEM/F12 for 72 h. Conditioned media were collected and concentrated 250-fold using 10 kDa Amicon filters (Millipore) at 3000 rpm for 15 min. N-linked glycosylation was assessed using PNGase F treatment (New England Biolabs) per manufacturer’s protocol, with samples incubated overnight at 37 °C.

Western blotting was performed using TGX Mini-PROTEAN SDS-PAGE gels (Bio-Rad) and transferred to 0.2 μm PVDF membranes (Bio-Rad, Cat# 1704156) using the Trans-Turbo Blot system. Blots were blocked with 5% milk, probed with primary antibodies overnight at 4 °C, and developed using ProteinID2.0 detection (Millipore). Chemiluminescent signal was captured on a Bio-Rad ChemiDoc MP imaging system. Antibodies used include anti-HA mouse clone 2-2.2.14 (ThermoFisher, Cat# 26183), preferred for detection from cell lysates due to the absence of nonspecific bands, and rabbit clone C29F4 (Cell Signaling, Cat# 3724), preferred for conditioned media samples due to more robust detection. Both primary antibodies were used at 1:5000 dilution; secondary antibodies were HRP-conjugated anti-mouse (ThermoFisher, Cat# 31430) and anti-rabbit (Southern Biotech, Cat# 4030–05) at 1:10,000 dilution.

### RNAi gene knockdown and gene expression analysis

Lentiviral shRNA constructs targeting *PRSS23* and *HTRA3* were obtained from the MISSION TRC shRNA library (Sigma-Aldrich). Multiple constructs were screened for knockdown efficiency, and the most effective were selected for experimental use: *PRSS23* KD1 (TRCN0000047041), KD2 (TRCN0000291207); *HTRA3* KD1 (TRCN0000075210), KD2 (TRCN0000075211). A NT shRNA control (NT; SCH001) was used in parallel. Lentiviral particles were produced in HEK293T cells using Lipofectamine 2000 or Lipofectamine 3000 (Thermo Fisher) and Opti-MEM (Thermo Fisher) according to the manufacturer’s instructions. Viral supernatants were collected at 24 and 48 h posttransfection, pooled, centrifuged at 3000 rpm to remove debris, filtered (0.2 μm PVDF), and aliquoted for storage at −80 °C.

For transduction, 1 × 10^6^ target cells were plated per 10-cm dish and exposed to 2 ml of thawed lentiviral supernatant supplemented with 10 μg/ml polybrene (Sigma-Aldrich). After 24 h, media were replaced with fresh culture medium containing 1 to 2 μg/ml puromycin for selection (exact concentration optimized per cell line). Cells were harvested after 72 h under selection for downstream assays or RNA analysis.

Total RNA was extracted using the RNeasy Mini Kit (Qiagen), and 1 μg of RNA was reverse transcribed using the High-Capacity cDNA Reverse Transcription Kit (Applied Biosystems). Gene expression was quantified by TaqMan-based qRT-PCR using 25 ng of cDNA for *HTRA3* (probes Hs00375742_m1, Hs00912756_m1) and 10 ng for *PRSS23* (probe Hs00970839_s1), normalized to *GAPDH* (probe Hs02786624_g1). Reactions were performed using the QuantStudio 7 Flex system and analyzed using QuantStudio 7 Flex software V1.7.2. The software was used to calculate relative quantification (RQ) using the 2^-ΔΔCt^ (Livak–Schmittgen) method based on mean Ct values, with uncertainty propagated in ΔCt space; RQ values are plotted as instrument-derived mean RQ ± error. For statistical analysis, a one-way ANOVA was used with the NT sample as the reference for comparisons in GraphPad Prism using Dunnett’s multiple comparisons test.

### CRISPR Cas-9

CRISPR-Cas9-mediated gene editing was outsourced to GenScript Biotech Co, Ltd. Single-guide RNA and DNA donor were designed, synthesized, and delivered to host cells together with Cas9 by electroporation. The process of cell engineering was performed in two phases. First, the 3×HA tag was inserted at the C terminus of *PRSS23* gene using gRNA 5′– GTCACCCCTCCCTACAATCC – 3′ flanked by 40-60 bp upstream and downstream sequences of the CRISPR/Cas9 binding locus. Cells were plated, screened, and a clone carrying the full-allelic 3 × HA modification was obtained and confirmed by Sanger sequencing. Upon completion of the first phase, the PRSS23_C term_3×HA homozygote clone was utilized for generating the S316A point mutation of the predicted catalytic serine (5′ – GATGCCCAGCCAGGGGCCAG – 3′). Each clone was tested negative for *mycoplasma* by MycoAlert Plus *Mycoplasma* Detection Kit from Lonza.

### Anoikis assay

Detachment-induced apoptosis (anoikis) was assayed using the RealTimeGlo Annexin V Apoptosis and Necrosis Assay kit (JA1011, Promega). Following lentiviral transduction and puromycin selection, cells were washed, trypsinized, and counted using the Countess automated cell counter (Thermo Fisher Scientific), then seeded (5000 per well) in 50 μl culture media with 50 μl of 2x Annexin V SmBiT/LgBiT with substrate, in 96-well uncoated white luminescence plates (3912, Costar). Plates were incubated at 37 °C while luminescence signal evolution was recorded up to 16 h using a Biotek synergy HTX system. The kit’s necrosis-associated fluorescence readout was monitored in parallel and remained minimal under these conditions. Each condition was tested with three or six technical replicates (as specified in figure legends), and each experiment was repeated a minimum of three times with consistent results. Statistical analysis was performed using two-way ANOVA in GraphPad Prism, with the NT at time 0 as the reference, employing Dunnett’s multiple comparisons test.

### Cell proliferation assay

Proliferation was assayed using the CellTiter-Glo 2.0 kit (G9242, Promega). Following lentiviral transduction and puromycin selection, cells were washed, trypsinized, and counted using the Countess automated cell counter (Thermo Fisher Scientific), then seeded (5000 per well) in cell culture media in 96-well plates. Cultures were incubated at 37 °C, 5% CO_2_ and 95% humidification for 16 h, and then equilibrated at RT for 30 min. CellTiter-Glo 2.0 reagent was equilibrated to RT, and then 100 μl of reagent was added to each well. Reactions were equilibrated another 10 min at RT with mixing on an orbital shaker, and then 100 μl of each reaction was transferred to a luminescence plate (3912, Costar), and luminescence signal was quantified using a GloMax 96 High Sensitivity Microplate Luminometer (9101-002, Promega). Each condition was assayed with three or six technical replicates (as specified in figure legends), and each experiment was repeated a minimum of three times with consistent results. Statistical analysis was performed using one-way ANOVA with Dunnett’s multiple comparisons test in GraphPad Prism.

### Animal models and bioluminescence imaging

All animal studies were conducted in accordance with protocols approved by the Mayo Clinic Institutional Animal Care and Use Committee (protocol A00005077-20-R23). Xenograft models were established as previously described (64). Female NOD/SCID mice (6–8 weeks old) were used for all experiments, and animals were evenly distributed across experimental groups to ensure comparable age and weight.

OVCA-420 and JHOC-5 cells were first transduced with lentiviral shRNA constructs targeting *PRSS23* (KD2) or NT, and knockdown efficiency was validated by qRT-PCR. Following puromycin selection, the cells were transduced with the pSin-luc lentiviral luciferase vector (65) to enable *in vivo* bioluminescence imaging.

For intraperitoneal xenografts, 5 × 10^6^ OVCA-420 cells or 5 × 10^5^ JHOC-5 cells in serum-free medium were injected into the lower right quadrant of the peritoneal cavity. To monitor tumor progression, mice received intraperitoneal injections of D-luciferin (100 mg/kg; GoldBio), followed by imaging after 8 min using the IVIS Spectrum 3D system (PerkinElmer/Caliper Life Sciences). Imaging was performed at baseline (Day 0), Day 1, and then weekly for up to 12 weeks for OVCA-420 or 7 weeks for JHOC-5, based on tumor growth kinetics and moribund endpoints.

Immediately prior to euthanasia, mice were reinjected with D-luciferin and imaged *in vivo*. Mice were euthanized by CO_2_ asphyxiation. After euthanasia, the abdominal cavity was opened, and *ex vivo* imaging was performed to visualize tumor dissemination. Tumor tissues and ascitic fluid (if present) were collected. In the OVCA-420 cohort, no ascites was detected.

Bioluminescence signal was quantified as ROI values using the IVIS analysis software. ROI values were log_10_-transformed prior to plotting and statistical testing. Within each xenograft model, longitudinal log_10_(ROI) data were analyzed by two-way repeated-measures ANOVA (time × condition) with Geisser-Greenhouse correction, followed by Šídák’s multiple comparisons test at each time point. *Ex vivo* endpoint ROI values were log_10_-transformed and compared between NT and KD groups using a Mann Whitney test. Analyses were performed in GraphPad Prism.

### RNA-seq and analysis

#### RNA sequencing

Total RNA from JHOC5 and OVCA-420 cells transduced with NT or *PRSS23* shRNAs (KD1 and KD2) was submitted to the Mayo Clinic sequencing core. Strand-specific poly(A) + RNA-seq libraries were prepared from high-quality RNA (RIN ≥ 8) according to the core facility’s standard protocol and sequenced on an Illumina platform to generate paired-end reads. Reads were aligned to the human reference genome and summarized as gene-level counts using Ensembl gene models, yielding a matrix of counts for 64,253 features, including Ensembl gene identifiers, gene symbols, genomic coordinates, and annotation of gene biotype (protein-coding, lncRNA, pseudogene, and others).

#### Preprocessing and normalization

All transcriptomic analyses were performed in R (v4.3.1). For each sample, raw counts were divided by the total library size and multiplied by one million to obtain counts per million (CPM). A pseudocount of one was added to each CPM, and values were transformed as log_2_(CPM + 1). Unless otherwise specified, analyses were restricted to genes annotated as “protein_coding” in the supplied annotation table, yielding 21,983 protein-coding genes. No additional filtering was applied, so genes with very low counts contributed little variance and did not materially influence PCA.

#### Principal component analysis

To compare global expression patterns between cell lines and knockdown conditions, we applied PCA to the log_2_-CPM matrix of protein-coding genes. For the initial PCA, gene expression values were mean-centered across all samples, and singular-value decomposition was used to compute principal components, with samples projected into the first two components. This analysis showed that PC1 primarily separated JHOC5 from OVCA-420, reflecting baseline histotype differences. To visualize knockdown-specific changes independent of cell-line effects, we generated a “cell line-adjusted” PCA by first subtracting, for each gene, its mean expression within each cell line separately and then repeating the PCA on the adjusted matrix.

#### Differential expression analysis and volcano plots

Differential expression was assessed separately within each cell line by comparing all *PRSS23* knockdown samples (KD1 and KD2 combined) to NT. For each protein-coding gene, we computed the mean CPM in NT and KD samples and derived the log_2_-fold change (KD *versus* NT) using a 0.5-count offset to stabilize ratios for low-abundance genes. Statistical testing was performed on log_2_-CPM values using a two-sided Welch’s *t* test, which does not assume equal variance between groups. Volcano plots were generated for each cell line by plotting log_2_-fold change against −log_10_(p) for all protein-coding genes.

#### Definition of the PRSS23 knockdown core signature and large heat map

To focus on transcriptional responses that were conserved between histotypes, we defined a “core” *PRSS23* knockdown signature across JHOC5 and OVCA-420. Gene-level differential expression tables for the two cell lines were merged using Ensembl gene identifiers. Among protein-coding genes, we selected those with absolute log_2_-fold change ≥ 1 in both JHOC5 and OVCA-420 and the same direction of change in both lines, irrespective of FDR. This yielded 193 genes, 70 upregulated and 123 downregulated in *PRSS23* knockdown relative to NT.

For the core-signature heat map, we extracted log_2_-CPM values for these 193 genes across all 12 RNA-seq samples. For each gene, values were scaled to z-scores by subtracting the mean and dividing by the standard deviation across samples. Hierarchical clustering of genes was performed using Euclidean distance and average linkage, and the clustered matrix was visualized as a heat map using a diverging color scale, with brown representing higher expression and purple lower expression relative to the gene mean. Samples were ordered by cell line and shRNA (JHOC5 NT, JHOC5 KD1, JHOC5 KD2, OVCA-420 NT, OVCA-420 KD1, and OVCA-420 KD2), and replicate identities were preserved.

#### Gene set enrichment and Hallmark gene set processing

To place the *PRSS23*-regulated genes in a pathway context, we conducted gene set enrichment analyses using the MSigDB Hallmark collection. Gene set enrichment was performed in R (v4.3.1) using the *fgsea* and *clusterProfiler* packages. For each cell line (JHOC5 and OVCA-420), we imported precomputed rank (RNK) files containing protein-coding genes and a signed statistic (signed −log_10_P derived from the *PRSS23* knockdown *versus* NT comparison). Genes with missing symbols were removed, and remaining genes were ordered in descending rank score to generate continuous ranked vectors. Hallmark gene sets (version v2025.1, HALLMARK_INFLAMMATORY_RESPONSE, HALLMARK_IL6_JAK_STAT3_SIGNALING, HALLMARK_TNFA_SIGNALING_VIA_NFKB, HALLMARK_EPITHELIAL_MESENCHYMAL_TRANSITION, HALLMARK_APICAL_JUNCTION, HALLMARK_PI3K_AKT_MTOR_SIGNALING, and HALLMARK_DNA_REPAIR) were downloaded as individual .gmt files and read with gmtPathways; all sets were combined into a single pathway list and converted to a TERM2GENE table. Enrichment was first quantified with fgsea (minSize = 15, maxSize = 500, 10,000 permutations), yielding normalized enrichment scores and FDR-adjusted *p* values for each pathway in each cell line; full results were exported as CSV tables. For visualization, the same ranked lists and TERM2GENE mapping were analyzed with clusterProfiler::GSEA (using the *fgsea* algorithm), and enrichment curves for selected Hallmark signatures (inflammatory response, IL6-JAK-STAT3, TNFα-NFκB, EMT, apical junction, and DNA repair) were plotted with enrichplot::gseaplot2 using custom R functions to overlay multiple pathways per cell line, increase line width, suppress legends, and display only the running enrichment score with the associated gene-rank tick marks.

#### Construction of focused Hallmark modules and mini heat maps

To visualize key pathways at gene resolution, we constructed four focused modules that combined Hallmark membership with inclusion in the *PRSS23* core signature. Gene sets were first intersected with the 193 core genes to identify overlapping members. For each biological axis, we selected seven representative genes that showed robust, concordant regulation in both lines and captured the diversity of Hallmark membership: a G2M checkpoint/DNA repair module (*AURKB, SLC7A5*, *TP53*, *MLH1*, *NEK6*, *CKS1B*, and *RPS3A*), a TNFα/NF-κB module (*NFKB1*, *SDC4*, *CXCL2*, *PLK2*, *BIRC3*, *CD83*, and *TNFAIP3*), an IL-6/inflammatory module (*HMOX1*, *IL6*, *EREG*, *SERPINE1*, *IL6R*, *CCL17*, and *PANX1*) and an EMT/adhesion module (*SERPINE1*, *FERMT2*, *ARPC3*, *SDC4*, *TGFBR3*, *AKT3*, and *EGFR*). Gene membership in each Hallmark set was determined directly from the .gmt files.

For each module, log_2_-CPM values were averaged across biological replicates within each condition to obtain six condition means: JHOC5 NT, JHOC5 KD1, JHOC5 KD2, OVCA-420 NT, OVCA-420 KD1, and OVCA-420 KD2. These means were scaled to z-scores per gene, and genes were hierarchically clustered using Euclidean distance and average linkage to define the row order for the mini heat maps. The resulting z-score matrices were plotted using the same brown-white-purple color scale and z-score range as the large core-signature heat map to allow direct visual comparison.

### Structural modeling

PRSS23 structural models were generated using AlphaFold3 *via* the AlphaFold Server (https://alphafoldserver.com/). In addition to the full-length protein, models were generated for the protease domain beginning at the predicted mature N terminus (Gln129, immediately C-terminal to the RRKR site) with three different C-terminal sequences: (*i*) the reference WT sequence with no C-terminal extension, (*ii*) a C-terminal LE spacer followed by 6×His tag, as incorporated into our recombinant *E*. *coli* construct, and (*iii*) a C-terminal 3×HA tag, as incorporated at the endogenous locus in CRISPR-engineered JHOC-5 cells. Predicted models were imported into PyMOL (Schrödinger, LLC) and structurally aligned to the WT reference model using backbone-based superposition. Structural comparisons focused on overall fold and conservation of the catalytic triad positioning.

### Recombinant PRSS23 construct design, expression, and purification

The human PRSS23 catalytic domain was codon optimized for *E. coli* and expressed as a recombinant fusion protein containing an N-terminal 6×His-SUMO tag followed by the PRSS23 catalytic domain and a C-terminal 6×His tag. The SUMO protease cleavage site was positioned between the SUMO tag and the PRSS23 catalytic domain to allow removal of the fusion tag after purification. A catalytic serine mutant (S316A) was generated by site-directed mutagenesis and expressed in parallel with the WT construct. Recombinant proteins were expressed in *E. coli* strain ArcticExpress (DE3) (Agilent Technologies, 230192) using Superior broth (Athena Enzyme Systems, 16-0121). Cultures were grown at 12 °C for 24 h, and then cells were lysed by sonication. Soluble PRSS23 was purified from clarified lysates using Ni^2+^-affinity chromatography under native conditions. Purified PRSS23 WT and S316A fusion proteins were incubated with SUMO protease (7.5 U/ml) at 4 °C overnight to remove the N-terminal SUMO tag or left untreated as controls. A parallel mock reaction included SUMO protease but no fusion protein substrate. Cleavage reactions were analyzed by SDS-PAGE followed by Coomassie staining. Bands corresponding to uncleaved fusion protein and cleaved (putatively active) PRSS23 catalytic domain were identified based on their expected molecular weights and their identity and sequence (including successful introduction of the S316A mutation) confirmed by LC-MS/MS protein ID. A BSA dilution series was run on each gel as a mass standard for densitometric estimation of the released PRSS23 protease domain band. Protein yields were insufficient for further purification of the PRSS23 catalytic domain, and so densitometric estimations were used to normalize protein input across peptide substrate assays.

### Protease activity assays using chromogenic substrates

Protease activity was assessed using chromogenic pNA substrates on an Agilent 8453 UV/Vis spectrophotometer in 100 μl cuvettes. Initial reaction rates were measured using each substrate at a final concentration of 200 μM in 20 mM Hepes pH 8.0, with WT PRSS23 or S316A catalytic domain at a final concentration of 300 nM, as estimated by gel densitometry of the released PRSS23 band. Reactions (final volume 100 μl) were initiated by addition of 10 μl enzyme (WT PRSS23 or S316A catalytic domain) to 90 μl of substrate/buffer mixture, and then absorbance at 405 nm was monitored for 5 min at 37 °C to calculate initial rates by linear regression. In the multisubstrate screen (Fig. 7*A*), WT PRSS23 was tested against all substrates, and the S316A mutant was tested under identical conditions against a subset that included substrates showing the highest apparent WT rates. Kallikrein (2 nM) was included as a positive control for cleavage of Z-FR-pNA. An equal volume aliquot of SUMO protease-only mock reaction (added in the place of PRSS23) and buffer blank served as negative controls. The following substrates were evaluated in kinetics assays: H-Ala-Phe-Pro-p-nitroanilide (Bachem, 4017695); N-Suc-Ala-Ala-Pro-Phe-p-NA (Sigma, S7388-100 mg); N-(p-Tosyl)-Gly-Pro-Lys-4-nitroanilide (Ac) (Sigma, T6140); Ac-Ile-Glu-Pro-Asp-pNA (Echelon Biosciences, 865-30); N-Succinyl-Ala-Ala-Pro-Leu-pNA (Sigma, S8511-10 mg); MeO-Suc-Arg-Pro-Tyr-pNA (Cayman, 27706); Suc-Ala-Ala-Pro-Met-pNA (Bachem, 4006760); Ac-Tyr-Val-Ala-Asp-pNA (Sigma, SML1424-5 mg); Bz-Ile-Glu-Gly-Arg-pNA (Cayman, 27567-5); Z-Phe-Arg-pNA (Bachem, 4009170); Suc-Ala-Ala-Pro-Arg-pNA (Bachem, 4017320); Z-Gly-Pro-Arg-p-nitroanilide (Sigma, C2276); MeOSuc-Ala-Ala-Pro-Val-p-nitroanilide (Bachem, 4004221); and N-Suc-Ala-Ala-pNA (Sigma, S4760-25 mg). Data points shown represent individual technical replicates.

### Activity-based probe labeling and immunoprecipitation

Conditioned media from JHOC-5 cells expressing WT or S316A mutant PRSS23-3×HA were clarified by centrifugation and 0.2 μm filtration, then concentrated 100-fold using 10 kDa Amicon filters (Sigma). Samples were labeled with ActivX TAMRA-FP probe (ThermoFisher) according to manufacturer instructions. Briefly, samples were adjusted to a final protein concentration of 2 mg/ml using IP buffer (25 mM Tris-HCl; 5% glycerol, 150 mM NaCl, 1 mM EDTA, 1% NP40, pH 7.4) and then treated with 2 μM TAMRA-FP probe for 30 min at 37 °C. A 100 μl aliquot was reserved for direct SDS-PAGE analysis. Remaining labeled media were buffer exchanged into TBST + 2% BSA using Zeba desalting spin columns (ThermoFisher) and immunoprecipitated as follows. Anti-HA magnetic beads (50 μl, Pierce, Cat# 88836) were incubated with the desalted conditioned media under rotation at 4 °C for 2 to 4 h. Beads were washed 3 to 5 times with IP buffer and eluted in 2× Laemmli sample buffer by boiling at 95 °C for 10 min. Eluted samples and flow through were analyzed by SDS-PAGE and immunoblotting. Gels were imaged by rhodamine fluorescence scanning and/or subjected to silver staining or immunoblotting. The probe labeling experiment was repeated multiple times with variations testing different probe concentrations (1–5 μM) and different labeling temperatures (RT, 37 °C, or 4 °C ON) with concordant results.

## Data availability

RNA-seq data have been deposited in Gene Expression Omnibus under accession GSE315399. All other data are contained within the manuscript and supplementary data.

## Supporting information

This article contains supporting information and cites references (66, 67, 68, 69, 70).

## Conflict of interest

The authors declare that they have no conflicts of interest with the contents of this article.
