# Supplementary material for: PRSS23 promotes ovarian cancer peritoneal dissemination independent of protease activity
Source: J Biol Chem. 2026 Apr 14;302(6):111450. doi: 10.1016/j.jbc.2026.111450 (PMC13194640; doi:10.1016/j.jbc.2026.111450)
Supplement: Supporting information [file mmc1.pdf]

Supplementary Tables and Figures for:

PRSS23 promotes ovarian cancer peritoneal dissemination independent of protease activity

Sharoon Akhtar[1,2], Matt Coban[1], Erin Miller[1], Alexandra Hockla[1], Eran Maina[1], Christine Mehner[1], Stefan Ilic[3], Niv Papo[3], Derek C. Radisky[1], Evette S. Radisky[1,*]

[1] Department of Cancer Biology, Mayo Clinic, Jacksonville, FL, USA.
[2] Mayo Clinic Graduate School of Biomedical Sciences, Mayo Clinic, Jacksonville, FL, USA
[3] Avram and Stella Goldstein-Goren Department of Biotechnology Engineering, Ben-Gurion University of the Negev, Beer-Sheva, Israel.

Materials Included:

Supplementary Table 1

Supplementary Table 2

Supplementary Figure S1

Supplementary Figure S2

Supplementary Figure S3

Supplementary Figure S4

Supplementary Figure S5

Supplementary Figure S6

Supplementary Figure S7

Supplementary Table S1. Prognostic associations for MEROPS-annotated S1 family serine proteases in ovarian cancer.

#	MEROPS ID	Peptidase	Gene	HR†	P-Value‡
1	S01.284	HtrA3 peptidase	*HTRA3*	1.58	4.70E-05
2	S01.291	TMPRSS12 peptidase	*TMPRSS12*	1.49	2.60E-04
3	S01.309	Serine Protease 23	*PRSS23*	1.43	5.80E-04
4	S01.231	urokinase-type plasminogen activator	*PLAU*	1.34	4.10E-05
5	S01.299	PRSS55 g.p. (*Homo sapiens*)	*PRSS55*	1.33	1.00E-02
6	S01.365	TMPRSS11B peptidase	*TMPRSS11B*	1.33	9.90E-03
7	S01.251	kallikrein-related peptidase 4	*KLK4*	1.32	1.20E-02
8	S01.286	Tysnd1 peptidase	*TYSND1*	1.29	3.40E-02
9	S01.132	mannan-binding lectin-associated serine peptidase-3	*MASP1*	1.28	2.00E-02
10	S01.198	mannan-binding lectin-associated serine peptidase 1	*MASP1*	1.28	2.00E-02
11	S01.277	HtrA1 peptidase (*Homo sapiens*-type)	*HTRA1*	1.24	3.90E-03
12	S01.153	elastase-1	*CELA1*	1.23	6.30E-02
13	S01.074	marapsin	*PRSS27*	1.23	5.00E-02
14	S01.019	corin	*CORIN*	1.23	2.30E-03
15	S01.215	coagulation factor VIIa	*F7*	1.21	8.90E-02
16	S01.531	protein FAM111B	*FAM111B*	1.21	7.60E-02
17	S01.081	kallikrein-related peptidase 15	*KLK15*	1.2	8.30E-02
18	S01.218	protein C (activated)	*PROC*	1.2	5.50E-03
19	S01.085	Mername-AA031 peptidase	*PRSS58*	1.19	9.50E-02
20	S01.087	TMPRSS13 peptidase	*TMPRSS13*	1.18	1.40E-01
21	S01.414	polyserase-2 unit 1	*PRSS36*	1.16	1.50E-01
22	S01.017	kallikrein-related peptidase 5	*KLK5*	1.16	6.20E-02
23	S01.015	tryptase beta	*TPSB2*	1.16	2.70E-02
24	S01.193	complement component activated C1s	*C1S*	1.15	5.80E-02
25	S01.191	complement factor D	*CFD*	1.14	4.40E-02
26	S01.232	t-plasminogen activator	*PLAT*	1.13	7.30E-02
27	S01.285	HtrA4 peptidase	*HTRA4*	1.12	2.90E-01
28	S01.258	trypsin-2 type A	*PRSS2*	1.12	1.20E-01
29	S01.147	granzyme H	*GZMH*	1.12	1.00E-01
30	S01.228	hepatocyte growth factor activator	*HGFAC*	1.11	1.40E-01
31	S01.278	HtrA2 peptidase	*HTRA2*	1.11	1.40E-01
32	S01.236	kallikrein-related peptidase 6	*KLK6*	1.08	2.60E-01
33	S01.127	cationic trypsin (*Homo sapiens*-type)	*PRSS1*	1.08	2.50E-01
34	S01.306	kallikrein-related peptidase 13	*KLK13*	0.95	4.30E-01
35	S01.021	DESC1 peptidase	*TMPRSS11E*	0.93	3.00E-01
36	S01.029	kallikrein-related peptidase 14	*KLK14*	0.93	2.70E-01
37	S01.300	kallikrein-related peptidase 7	*KLK7*	0.92	4.50E-01
38	S01.159	prostasin	*PRSS8*	0.92	2.70E-01

39	S01.192	complement component activated C1r	*C1R*	0.92	2.40E-01
40	S01.174	mesotrypsin	*PRSS3*	0.92	2.30E-01
41	S01.152	chymotrypsin B	*CTRB1*	0.91	1.80E-01
42	S01.011	testisin	*PRSS21*	0.91	1.60E-01
43	S01.146	granzyme K	*GZMK*	0.91	1.60E-01
44	S01.154	pancreatic endopeptidase E	*CELA3A*	0.9	1.50E-01
45	S01.302	matriptase	*ST14*	0.9	1.20E-01
46	S01.020	kallikrein-related peptidase 12	*KLK12*	0.89	2.60E-01
47	S01.298	trypsin C	*TRY6*	0.89	1.30E-01
48	S01.155	pancreatic elastase II	*CELA2A*	0.89	1.20E-01
49	S01.162	kallikrein-related peptidase 3	*KLK3*	0.89	1.20E-01
50	S01.205	pancreatic endopeptidase E form B	*CELA3B*	0.89	7.00E-02
51	S01.224	hepsin	*HPN*	0.88	6.80E-02
52	S01.161	kallikrein-related peptidase 2	*KLK2*	0.88	6.60E-02
53	S01.028	tryptase gamma 1	*TPSG1*	0.88	6.40E-02
54	S01.252	prosemin	*PRSS22*	0.88	4.60E-02
55	S01.217	thrombin	*F2*	0.88	4.50E-02
56	S01.157	chymotrypsin C	*CTRC*	0.87	4.70E-02
57	S01.079	transmembrane peptidase, serine 3	*TMPRSS3*	0.87	4.60E-02
58	S01.246	kallikrein-related peptidase 10	*KLK10*	0.87	4.20E-02
59	S01.133	cathepsin G	*CTSG*	0.86	4.50E-02
60	S01.313	spinesin	*TMPRSS5*	0.86	3.50E-02
61	S01.247	epitheliasin	*TMPRSS2*	0.85	1.70E-01
62	S01.213	coagulation factor XIa	*F11*	0.85	1.70E-02
63	S01.160	kallikrein 1	*KLK1*	0.85	1.40E-02
64	S01.047	human airway trypsin-like peptidase	*TMPRSS11D*	0.84	2.10E-02
65	S01.134	myeloblastin	*PRTN3*	0.84	1.40E-02
66	S01.135	granzyme A	*GZMA*	0.84	1.40E-02
67	S01.140	chymase (*Homo sapiens*-type)	*CMA1*	0.84	1.00E-02
68	S01.307	kallikrein-related peptidase 9	*KLK9*	0.83	6.50E-02
69	S01.214	coagulation factor IXa	*F9*	0.83	9.50E-03
70	S01.211	coagulation factor XIIa	*F12*	0.83	6.80E-03
71	S01.308	matriptase-2	*TMPRSS6*	0.83	6.00E-03
72	S01.034	transmembrane peptidase, serine 4	*TMPRSS4*	0.83	5.80E-03
73	S01.199	complement factor I	*CFI*	0.83	5.00E-03
74	S01.233	plasmin	*PLG*	0.82	6.00E-02
75	S01.189	complement component C1r-like peptidase	*C1RL*	0.82	9.50E-03
76	S01.206	pancreatic elastase IIB	*CELA2B*	0.82	8.60E-03
77	S01.131	elastase-2	*ELANE*	0.82	5.40E-03
78	S01.194	complement component C2a	*C2*	0.82	2.60E-03
79	S01.196	complement factor Bb	*CFB*	0.82	2.60E-03
80	S01.229	mannan-binding lectin-associated serine peptidase 2	*MASP2*	0.81	4.10E-03

81	S01.530	protein FAM111A	*FAM111A*	0.81	1.90E-03
82	S01.256	chymopasin	*CTRL*	0.81	1.40E-03
83	S01.244	kallikrein-related peptidase 8	*KLK8*	0.8	3.80E-02
84	S01.212	plasma kallikrein	*KLKB1*	0.8	1.10E-03
85	S01.054	tryptase delta 1 (*Homo sapiens*)	*TPSD1*	0.8	1.00E-03
86	S01.216	coagulation factor Xa	*F10*	0.8	8.00E-04
87	S01.139	granzyme M	*GZMM*	0.8	4.90E-04
88	S01.075	tryptase-6	*PRSS33*	0.79	3.60E-02
89	S01.237	neurotrypsin	*PRSS12*	0.79	1.50E-03
90	S01.223	acrosin	*ACR*	0.79	1.30E-03
91	S01.033	factor VII-activating peptidase	*HABP2*	0.79	8.80E-04
92	S01.010	granzyme B (*Homo sapiens*-type)	*GZMB*	0.78	9.60E-04
93	S01.143	tryptase alpha	*TPSAB1*	0.78	7.30E-04
94	S01.257	kallikrein-related peptidase 11	*KLK11*	0.77	2.10E-04
95	S01.072	matriptase-3	*TMPRSS7*	ND§	ND§
96	S01.078	ovochymase-1 domain 1	*OVCH1*	ND	ND
97	S01.088	Mername-AA038 peptidase	*PRSS47P*	ND	ND
98	S01.156	enteropeptidase	*TMPRSS15*	ND	ND
99	S01.318	marapsin-2	*PRSS38*	ND	ND
100	S01.319	neutrophil serine peptidase 4	*PRSS57*	ND	ND
101	S01.320	ovochymase-2	*OVCH2*	ND	ND
102	S01.321	transmembrane peptidase, serine 11F	*TMPRSS11F*	ND	ND
103	S01.322	ovochymase 1 domain 1	*OVCH1*	ND	ND
104	S01.325	epidermis-specific SP-like putative peptidase	*PRSS48*	ND	ND
105	S01.326	testis serine peptidase 5	*PRSS45P*	ND	ND
106	S01.327	Mername-AA258 peptidase	*PRSS41*	ND	ND
107	S01.362	testis serine peptidase 2 (human-type)	*PRSS42P*	ND	ND
108	S01.414	polyserase-3 unit 1	*PRSS53*	ND	ND
109	S01.375	polyserase-3 unit 2	*PRSS53*	ND	ND
110	S01.376	peptidase similar to tryptophan/serine protease	*PRSS51*	ND	ND
111	S01.487	hCG2041452-like protein	*PRSS43*	ND	ND
112	S01.514	PRSS56 peptidase	*PRSS56*	ND	ND
113	S01.292	HAT-like putative peptidase 2	*TMPRSS11A*	ND	ND

† HR (OS), hazard ratio for overall survival (high vs low expression); HR > 1 indicates worse OS in the high-expression group.
‡ P value, log-rank test.
§ ND, no data (gene not represented in the KMplotter ovarian cancer datasets).

Supplementary Table S2: Prognostic associations for additional genes implicated as transcriptional biomarkers in ovarian cancer.

Protein	Gene	HR[†,§]	P-Value[‡,§]	citation
Histamine N-methyltransferase	*HNMT*	1.21	0.093	(66)
3-oxo-5-alpha-steroid 4-dehydrogenase 1	*SRD5A1*	1.12	0.094	(66)
Procollagen-lysine,2-oxoglutarate 5-dioxygenase 2	*PLOD2*	1.17	0.017	(66)
Breast cancer type 2 susceptibility protein	*BRCA2*	1.17	0.028	(67)
Creatine kinase B-type	*CKB*	1.18	0.022	(68)
Forkhead box protein M1	*FOXM1*	1.16	0.057	(69)
Claudin-3	*CLDN3*	1.24	0.002	(70)
Urokinase-type plasminogen activator	*PLAU*	1.34	0.000041	(18)

[†] HR (OS), hazard ratio for overall survival (high vs low expression); HR > 1 indicates worse OS in the high-expression group.
[‡] P value, log-rank test.
[§] Calculated from KMPlotter using the same settings as used for Table S1.

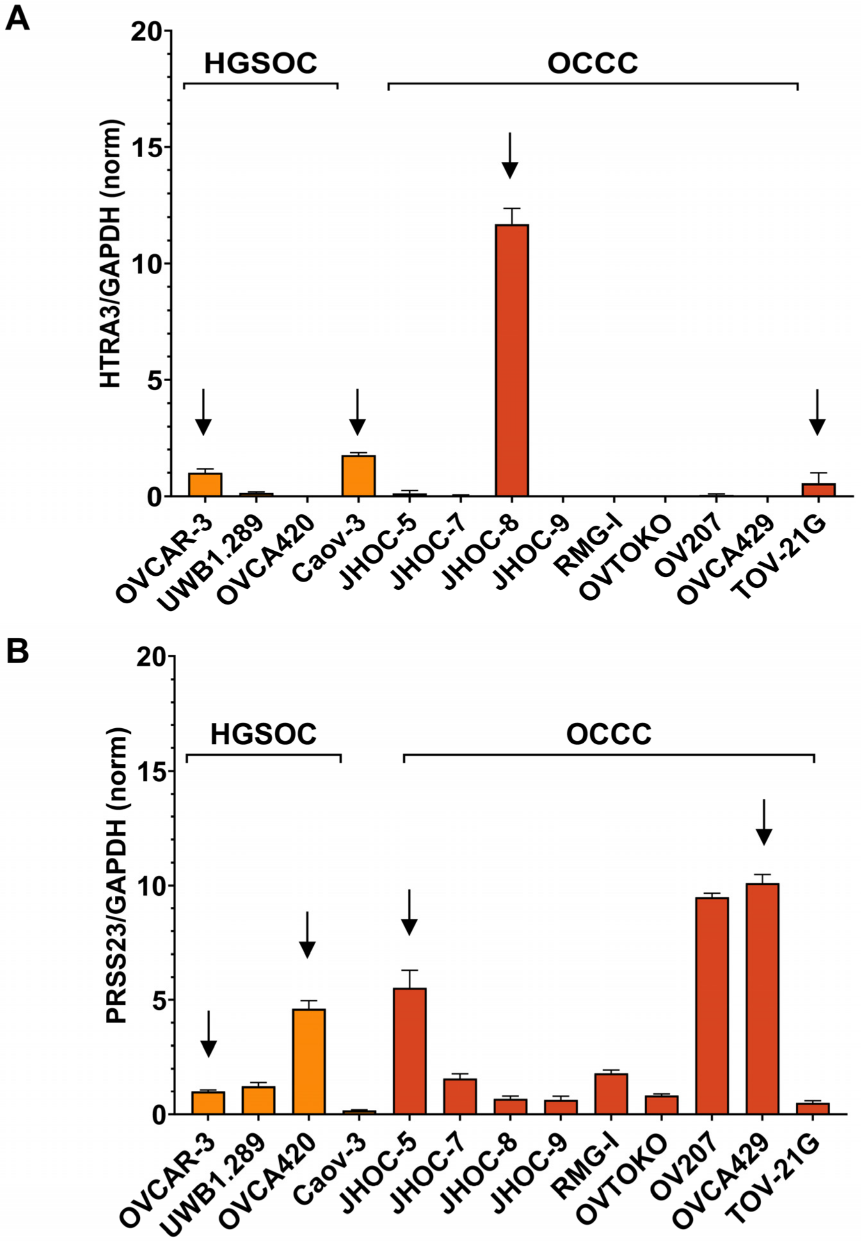

Supplementary Figure S1. Baseline expression of HTRA3 and PRSS23 across ovarian cancer cell line models. (A) HTRA3 mRNA expression across the ovarian cancer cell line panel measured by qRT-PCR and normalized to GAPDH. N = 3 per condition. Cell lines are grouped by histotype (HGSOC vs OCCC) as indicated. Arrows denote the four cell lines selected for HTRA3 knockdown studies. **(B)** PRSS23 mRNA expression across the same cell line panel measured by qRT-PCR and normalized to GAPDH. N = 3 per condition. Arrows denote the four cell lines selected for PRSS23 knockdown studies.

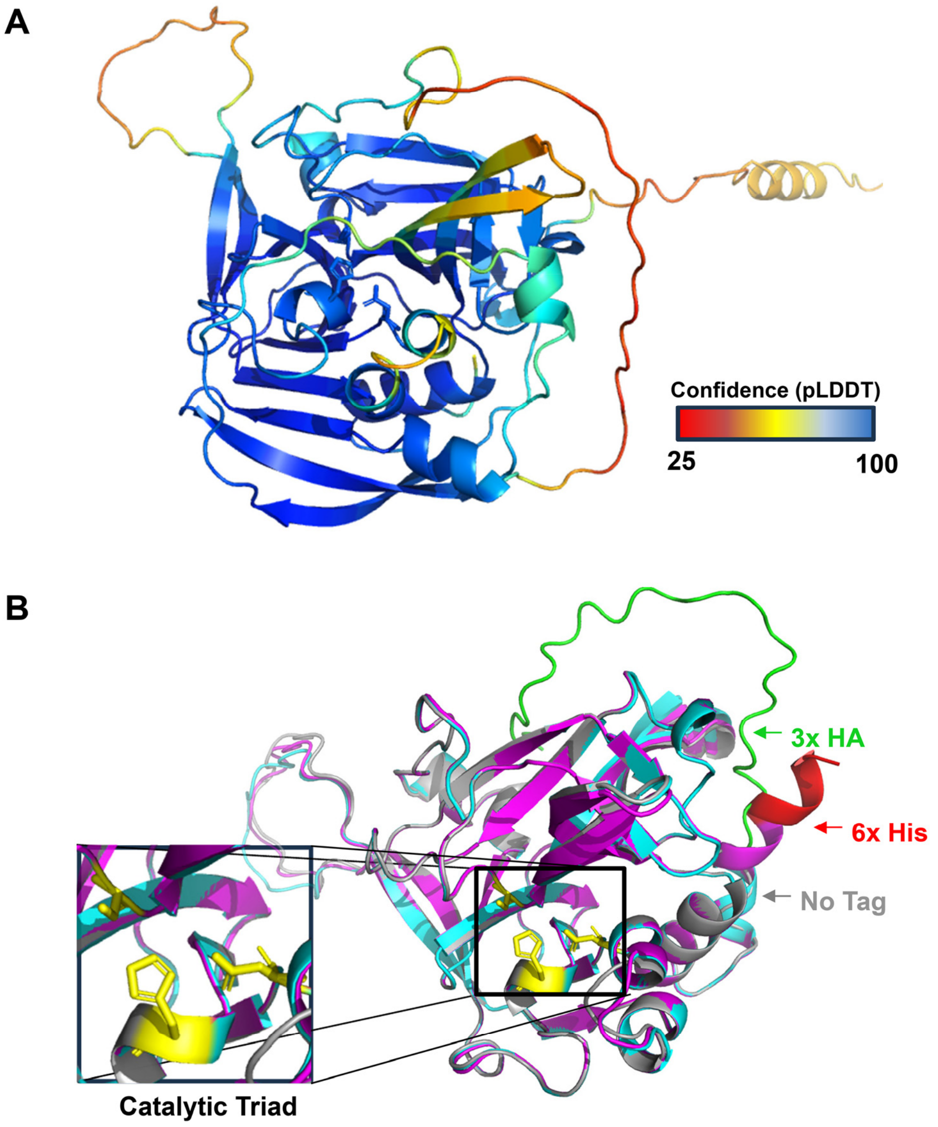

Supplementary Figure S2. AlphaFold models predict a high-confidence PRSS23 serine protease domain and minimal impact of C-terminal epitope tags. ***A***, AlphaFold-predicted structure of full-length human PRSS23 colored by predicted local confidence score (pLDDT; *blue* = higher confidence, *red* = lower confidence). The C-terminal S1 serine protease homology domain is predicted with higher confidence, including the region surrounding the conserved Ser-His-Asp triad (shown as *sticks*). In contrast, the N-terminal signal sequence and prodomain, along with several extended loop insertions within the protease homology domain, are predicted with lower confidence. ***B***, Superposition of AlphaFold-predicted PRSS23 serine protease domain models without tag (*gray*) or containing short C-terminal epitope tag extensions (6×His model shown in *magenta* with tag in *red*; 3×HA model shown in *cyan* with tag in *green*). The protease domain core and catalytic triad geometry are predicted to be superimposable across models, with tag sequences extending from the C-terminus without appreciable perturbation of the catalytic core.

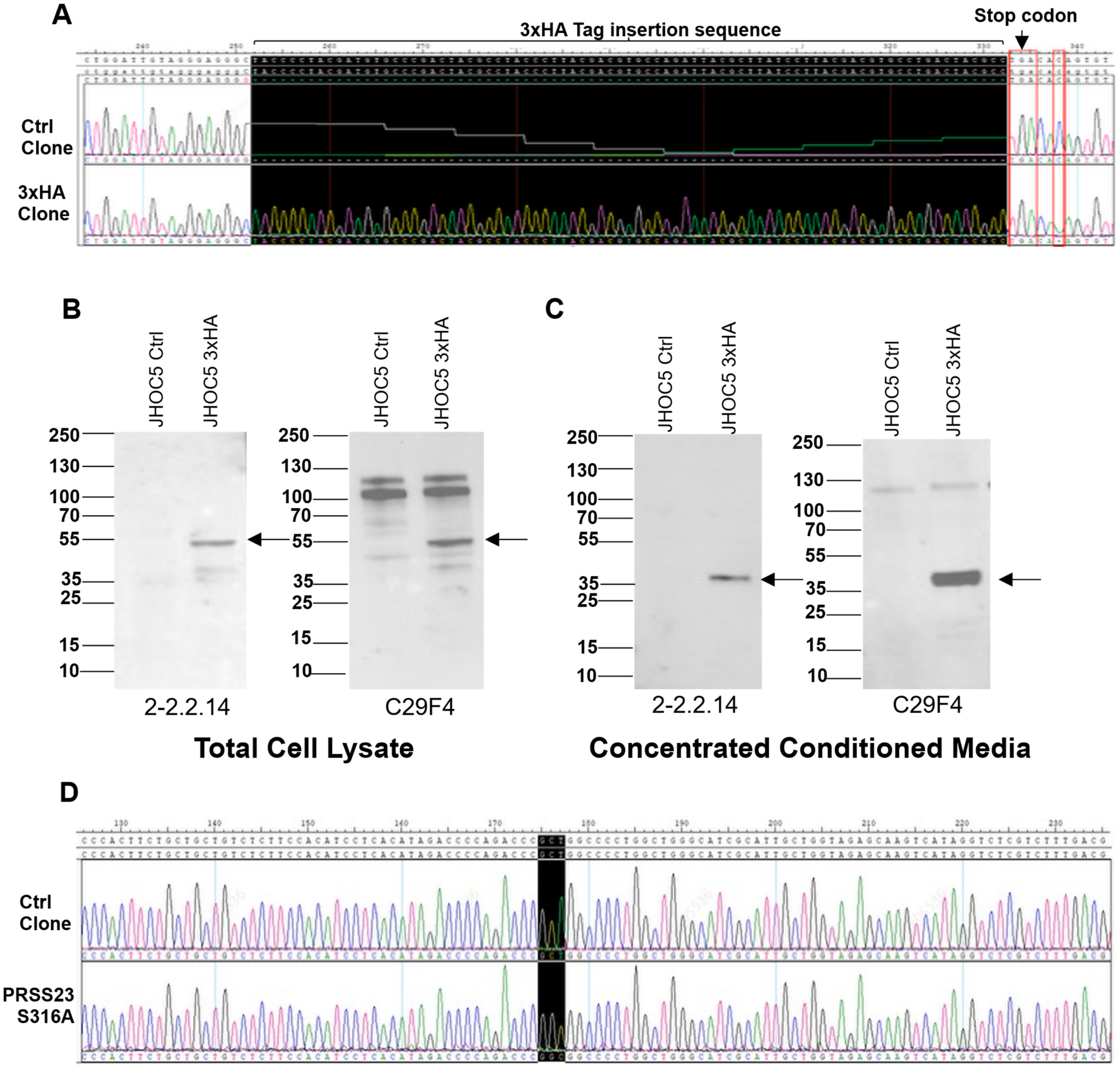

Supplementary Figure S3. Validation of PRSS23-3×HA knock-in and Ser316Ala editing in JHOC-5 cells. (A) Sanger sequencing chromatograms of clonal isolates confirmed CRISPR/Cas9-mediated integration of the C-terminal 3×HA tag at the endogenous PRSS23 locus, with no detectable unedited allele. (B,C) Anti-HA immunoblot detection of PRSS23-3×HA in total cell lysates **(B)** and concentrated conditioned media **(C)** from control and HA-tagged JHOC-5 cells using ThermoFisher clone 2-2.2.14 (left) and Cell Signaling clone C29F4 (right). Arrows indicate bands corresponding to detected PRSS23-3×HA species. Note that the left panel of (B) is reproduced from main Fig. 6C and the right panel of (C) is reproduced from main Fig. 6D. **(D)** Sanger sequencing chromatograms confirm precise introduction of the Ser316Ala (S316A) substitution at the endogenous PRSS23-3×HA locus, with no detectable unedited allele in the mutant clone.

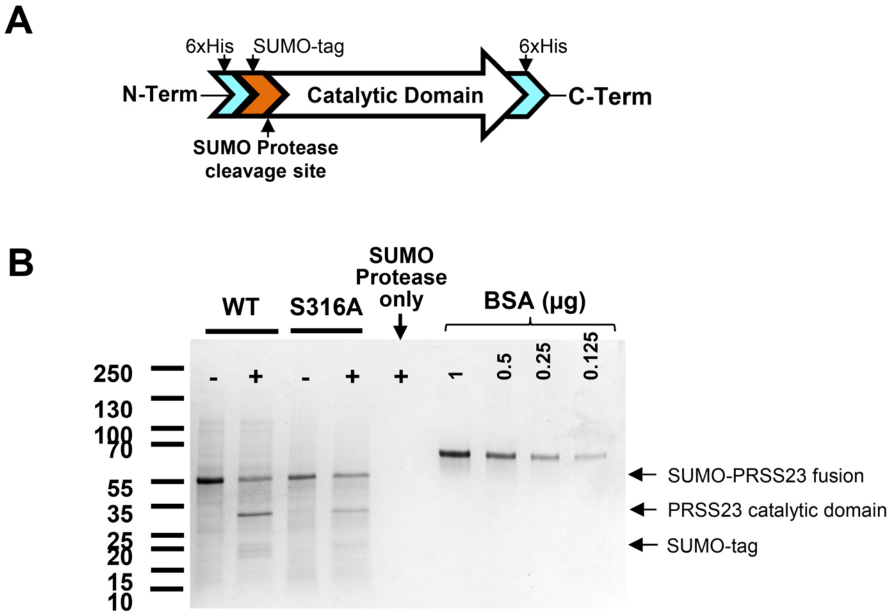

Supplementary Figure S4. Recombinant SUMO-PRSS23 protease domain construct and representative SUMO protease cleavage reaction. *A,* Schematic of the recombinant PRSS23 protease-domain expression construct, produced as an N-terminal 6×His-SUMO fusion with a C-terminal 6×His-tag. The SUMO protease cleavage site is indicated. *B,* Representative SDS-PAGE gel showing cleavage of recombinant WT and PRSS23-S316A SUMO fusion proteins by a catalytic quantity of SUMO protease (not visible on gel). "-" and "+" indicate reactions performed without or with SUMO protease, respectively. A "SUMO protease only" control lane represents a mock-digestion reaction with SUMO protease at identical concentration in the absence of SUMO-PRSS23 substrate, as used for SUMO protease control assays in Fig. 7B. A BSA dilution series (1, 0.5, 0.25, 0.125 µg) is included as a mass standard for densitometric estimation of the released PRSS23 protease domain band, the approach used to normalize protein input across peptide substrate assays in Fig. 7A-B.

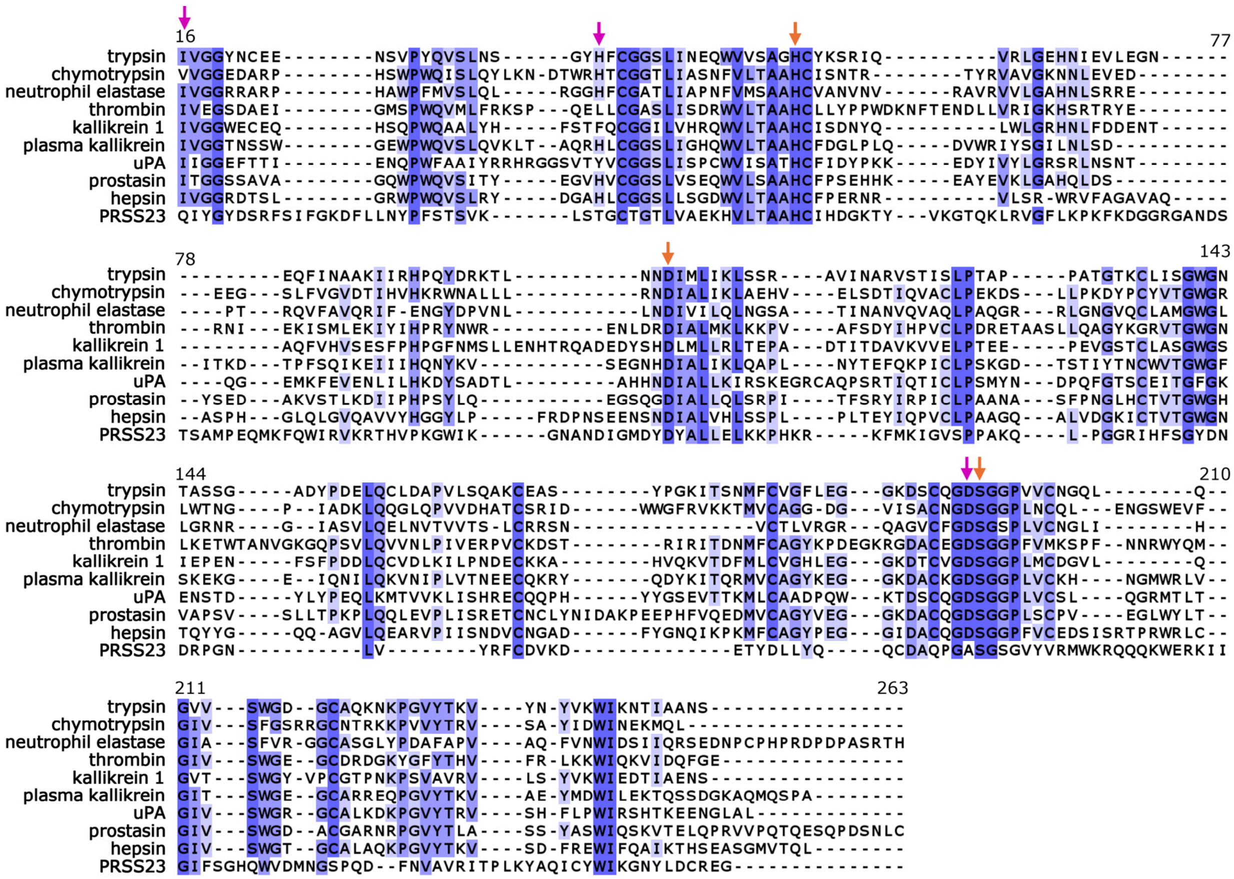

Supplementary Figure S5. PRSS23 serine protease homology domain is highly diverged from active S1 proteases. Multiple sequence alignment of selected human S1 serine protease catalytic domains and PRSS23. Magenta arrows indicate positions corresponding to the zymogen activation switch (including the Ile16 and Asp194 positions, chymotrypsin numbering), and orange arrows indicate catalytic triad residues. Despite preservation of the catalytic triad, PRSS23 diverges substantially from active family members and contains multiple insertions/deletions, consistent with remodeling of protease domain surface loops that may support nonproteolytic functions. Alignment was created in Clustal Omega and visualized in JalView colored by BLOSUM62 conservation score. Sequences were obtained from UniProt: trypsin P07477; chymotrypsin Q99895; neutrophil elastase P08246; thrombin P00734; kallikrein 1 P06870; plasma kallikrein P03952; uPA P00749; prostasin Q16651; hepsin P05981; PRSS23 O95084.

Supplementary Figure S6A

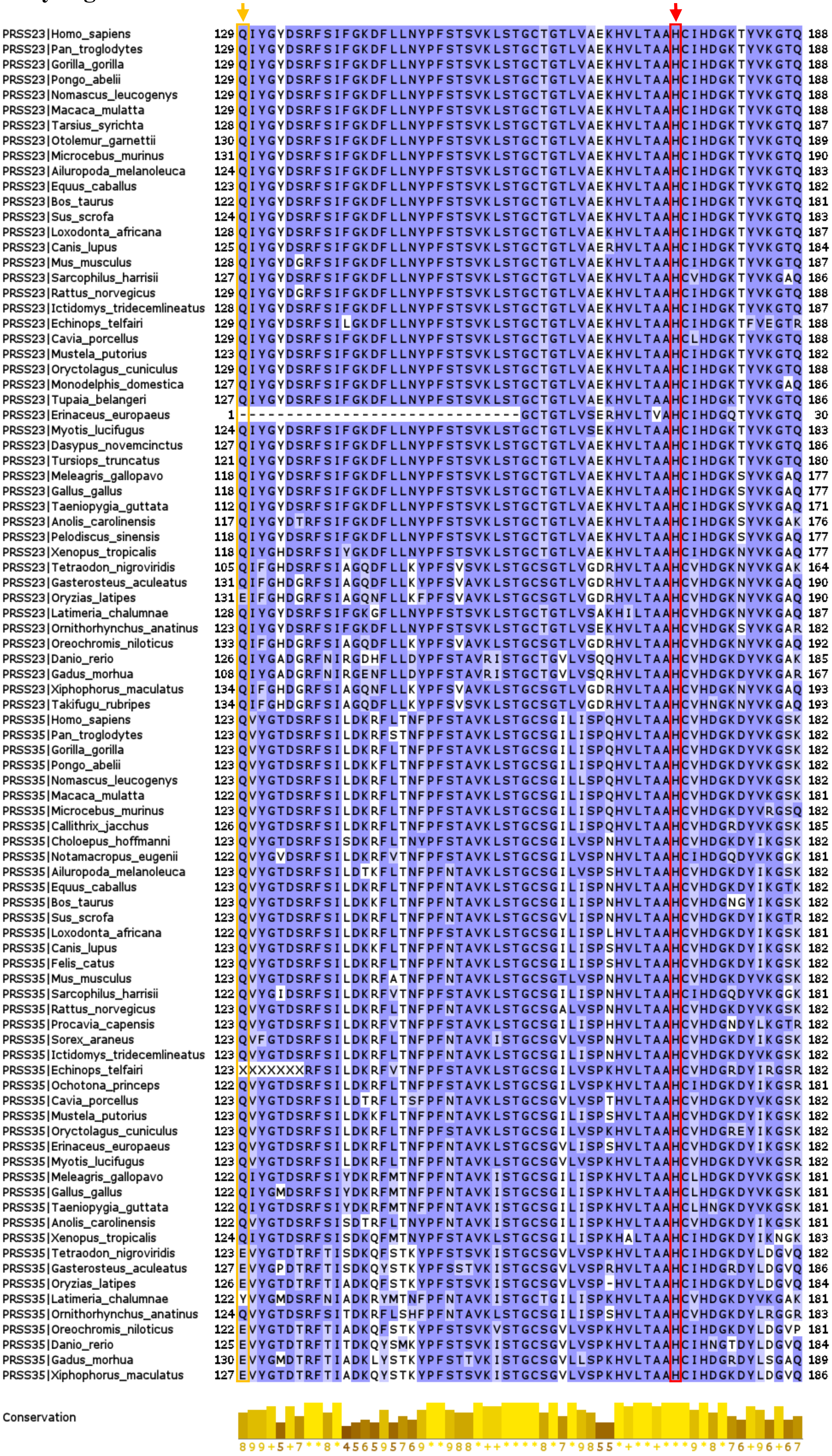

Supplementary Figure S6B

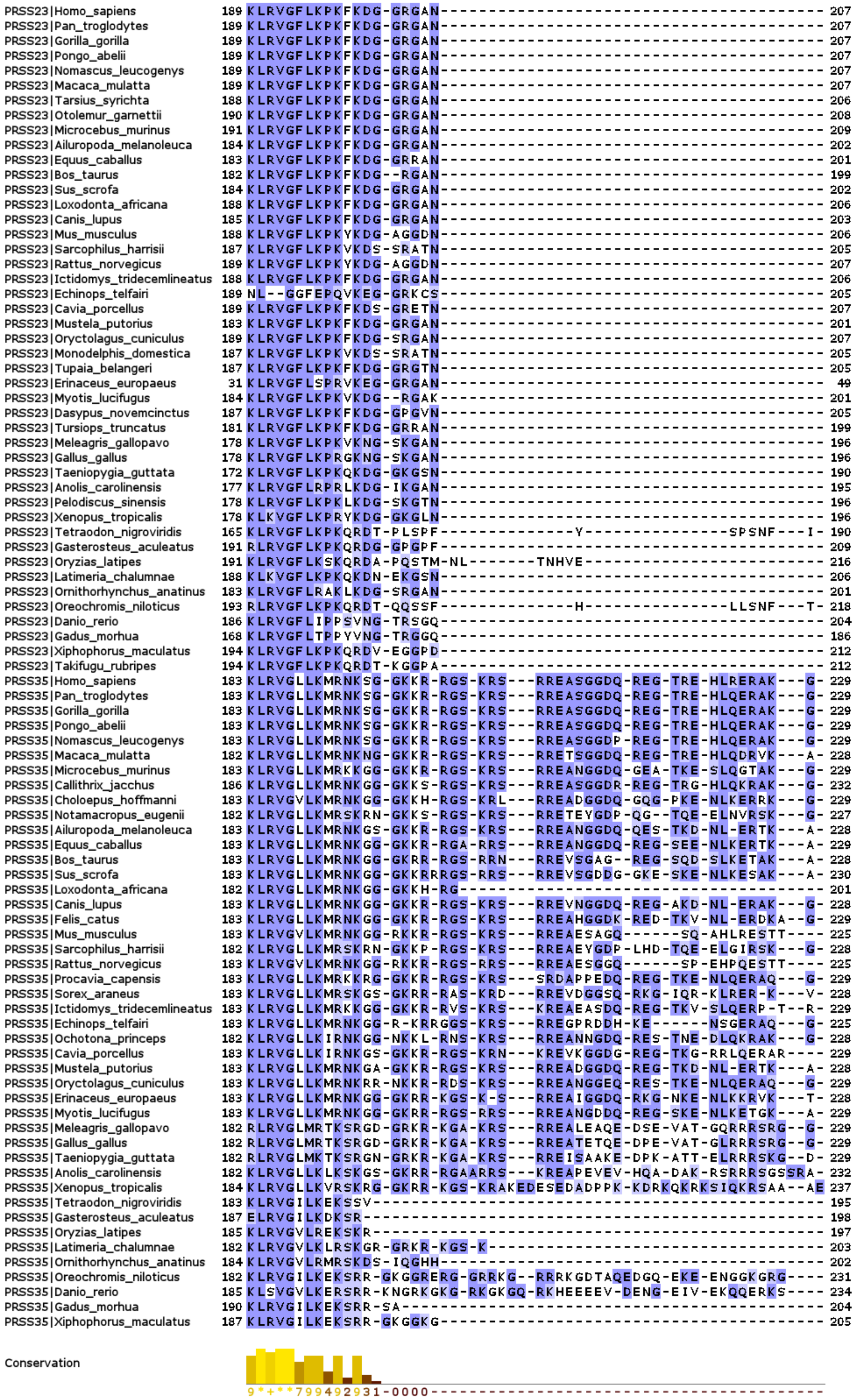

Supplementary Figure S6C

```
PRSS23|Homo_sapiens                208 --------DSTSAMPEQMKFQWIRVKRTHVPKGWIKG-NANDIGMDYDYALLELKKPHKR 258
PRSS23|Pan_troglodytes             208 --------DSTSAMPEKMKFQWIRVKRTHVPKGWIKG-NANDIGMDYDYALLELKKPHKR 258
PRSS23|Gorilla_gorilla             208 --------DSTSAMPEKMKFQWIRVKRTHVPKGWIKG-NANDIGMDYDYALLELKKPHKR 258
PRSS23|Pongo_abelii                208 --------DSTSAIPEKMKFQWIRVKRTHVPKGWIKG-NANDIGMDYDYALLELKKPHKR 258
PRSS23|Nomascus_leucogenys         208 --------DSTSAMPEKMKFQWIRVKRTHVPKGWIKG-NANDIGMDYDYALLELKKPHKR 258
PRSS23|Macaca_mulatta              208 --------DSTSAMPEKMKFQWIRVKRTHVPKGWIKG-NANDIGMDYDYALLELKKPHKR 258
PRSS23|Tarsius_syrichta            207 --------DSSSAMPEKMKFQWIRVKRTHVPKGWIKG-NANDIGMDYDYALLELKKPHKR 257
PRSS23|Otolemur_garnettii          209 --------NSSSAIPEKMKFQWIRVKRTHVPKGWIKG-NANDIGMDYDYALLELKKPHKR 259
PRSS23|Microcebus_murinus          210 --------DSSSAVPEKMKFQWIRVKRTHVPKGWIKG-NANDIGMDYDYALLELKKPHKR 260
PRSS23|Ailuropoda_melanoleuca      203 --------SSSSGVPEKMKFQWIRVKRTHVPKGWIKG-NANDIGMDYDYALLELKKPHKR 253
PRSS23|Equus_caballus              202 --------DSSSAVPEKMKFQWIRVKRTHVPKGWIKG-NANDIGMDYDYALLELKKPHKR 252
PRSS23|Bos_taurus                  200 --------DSHSALPEKMKFQWIRVKRTHVPKGWIKG-NANDIGMDYDYALLELKKPHKR 250
PRSS23|Sus_scrofa                  203 --------NSSSAVPEKMKFQWIRVKRTHVPKGWIKG-NANDIGMDYDYALLELKKPHKR 253
PRSS23|Loxodonta_africana          207 --------SSNSAIPEKMKFQWIRVKRTHVPKGWIKG-NANDIGMDYDYALLELKKPHKR 257
PRSS23|Canis_lupus                 204 --------SSSSAIPEKMKFQWIRVKRTHVPKGWIKG-NANDIGMDYDYALLELKKPHKR 254
PRSS23|Mus_musculus                207 --------SSSSAMPDKMKFQWIRVKRTHVPKGWIKG-NANDIGMDYDYALLELKKPHKR 257
PRSS23|Sarcophilus_harrisii        206 --------TSSSAIPDKMKFQWIRVKRTHVPKGWIKG-NANDIGMDYDYALLELKKPHKR 256
PRSS23|Rattus_norvegicus           208 --------SSSSALVEKMKFQWIRVKRTHVPKGWIKG-NANDIGMDYDYALLELKKPHKR 258
PRSS23|Ictidomys_tridecemlineatus  207 --------SSSSALPEKMKFQWIRVKRTHVPKGWIKG-NANDIGMDYDYALLELKKPHKR 257
PRSS23|Echinops_telfairi           206 ------------SVPDK-MFLWFRLKRTHVPKGWIKG-NANDIGMDYDYALLELKKPHKR 251
PRSS23|Cavia_porcellus             208 --------TSNSATPEKMKFQWIRVKRTHVPKGWIKG-NANDIGMDYDYALLELKKPHKR 258
PRSS23|Mustela_putorius            202 --------SSSSAGPEKMKFQWIRVKRTHVPKGWIKG-NANDIGMDYDYALLELKKPHKR 252
PRSS23|Oryctolagus_cuniculus       208 --------NSSSAIPEKMKFQWIRVKRTHVPKGWIKG-NANDIGMDYDYALLELKKPHKR 258
PRSS23|Monodelphis_domestica       206 --------ISKSAIPDKMKFQWIRVKRTHVPKGWIKG-NANDIGMDYDYALLELKKPHKR 256
PRSS23|Tupaia_belangeri            206 --------ESNPAMPEKMKFQWIRVKRTHVPKGWIKG-NANDIGMDYDYALLELKKPHKR 256
PRSS23|Erinaceus_europaeus          50 --------TSGPGRPPQTRFQWIRVKRTHVPKGWIKG-SANDIGMDYDYALLELRKPHRR 100
PRSS23|Myotis_lucifugus            202 --------NSSAAVPEKMKFQWIRVKRTHVPKGWIKG-NANDIGMDYDYALLELKKLHKR 252
PRSS23|Dasypus_novemcinctus        206 --------GSSPAGPEKMKFQWIRVKRTHVPKGWIKG-NANDIGMDYDYALLELKKPHRR 256
PRSS23|Tursiops_truncatus          200 --------DSSSAVPEKMKFQWIRVKRTHVPKGWIKG-NANDIGMDYDYALLELKKPHKR 250
PRSS23|Meleagris_gallopavo         197 --------ITGSAMPEKMKFQWIRVKRTHVPKGWIKG-NANDIGMDYDYALLELKKPHKR 247
PRSS23|Gallus_gallus               197 --------ITSSAMPEKMKFQWIRVKRTHVPKGWIKG-NANDIGMDYDYALLELKKPHKR 247
PRSS23|Taeniopygia_guttata         191 --------ITNSAMPEKMKFQWIRVKRTHVPKGWIKG-NANDIGMDYDYALLELKKPHKR 241
PRSS23|Anolis_carolinensis         196 --------VSSSPDPHKMKFQWIRVKRTHVPKGWIKG-NANDIGMDYDYALLELKKPHKR 246
PRSS23|Pelodiscus_sinensis         197 --------ITSSTMPEKMKFQWIRVKRTHVPKGWIKG-NANDIGMDYDYALLELKKPHKR 247
PRSS23|Xenopus_tropicalis          197 --------QTNPRIPEKMKFQWIRVKRTHVPKGWIKG-NANDIGMDYDYALLELKKPHKR 247
PRSS23|Tetraodon_nigroviridis      191 NRVGGGPTPYVPPTNSKMKFQWIRAKRTHIPKGWIKG-NANEIGMDYDYALLELKKPHKR 249
PRSS23|Gasterosteus_aculeatus      210 ----------GPPTDDEMKFQWIRAKRTHVPKGWIKG-NANDIGMDYDYALLELKKSHKR 258
PRSS23|Oryzias_latipes             217 ----GGPALYTPPTKDKMKFQWIRVKRTHVPKGWIKG-NANDIGMDFDYALLELKKPHKR 271
PRSS23|Latimeria_chalumnae         207 --------QTSTLHPEKMKFQWMRVKRTHVPKGWIKG-NANDIGMDYDYALLELKKPHKR 257
PRSS23|Ornithorhynchus_anatinus    202 --------VSLSSVPERAKFQWIRVKRTHVPKGWIQG-NANDIGMDYDYALLELKKPHKR 252
PRSS23|Oreochromis_niloticus       219 NSVEGGTSPYAPPTSDKMKFQWIRAKRTHVPKGWIKG-NANDIGMDYDYALLELKKPHKR 277
PRSS23|Danio_rerio                 205 ------------APVKKPLVRWVRVKRTRVPKGWIQG--PQEVSMDFDYALLELRWPHRR 250
PRSS23|Gadus_morhua                187 ------------SDPRKPLVRWVRVKRTRVPKGWIQG--PQEVSMDFDYALLELRWPHRR 232
PRSS23|Xiphophorus_maculatus       213 --------QYSPPANDKMKFQWIRAKRTHVPKGWIKG-NANDIGMDYDYALLELKKPHKR 263
PRSS23|Takifugu_rubripes           213 --------PYVPPTNTKMKFQWIRAKRTHVPKGWIKG-NANEIGMDYDYALLELKKPHKR 263
PRSS35|Homo_sapiens                230 GRRRKKSGRGQRIAEGRPSFQWTRVKNTHIPKGWARG-GMGDATLDYDYALLELKRAHKK 288
PRSS35|Pan_troglodytes             230 GRRRKQSGRGQRVAEGRPSFQWTRVKNTHIPKGWARG-GMGDAALDYDYALLELKRAHKK 288
PRSS35|Gorilla_gorilla             230 GRRRKQSGRGQRIAEGRPSFQWTRVKNTHIPKGWARG-GMGDAALDYDYALLELKRAHKK 288
PRSS35|Pongo_abelii                230 GRRRKQSGRGQRVAEGRPSFQWTRVKNTHIPKGWARG-GMGDAALDYDYALLELKRAHKK 288
PRSS35|Nomascus_leucogenys         230 GRSRKQAGRGQRLAEGRPSFQWTRVKNTHIPKGWARG-GLGDAALEYDYALLELKRAHKK 288
PRSS35|Macaca_mulatta              229 GRRRKQSGGGQRVSEGRPSFRWTRVKNTHIPKGWARG-GMGDAALDYDYALLELKRAHKK 287
PRSS35|Microcebus_murinus          230 GRRRKASGRGRRVAEGRPSFQWTRVKNTHIPKGWARG-GREDAALDYDYALLELKRAHKK 288
PRSS35|Callithrix_jacchus          233 GRRRKQSGRGQRISEGRPSFQWTRVKNTHIPKGWARG-GSEDAALDYDYALLELKRAHKK 291
PRSS35|Choloepus_hoffmanni         230 RRRE--SGRRQQGVEGRPSFQWTRVKNTHIPAGWARA-GMGDAALDYDYALLELKRAHRK 286
PRSS35|Notamacropus_eugenii        228 RRRRKGLGKGQRGSEGQPSFQWTRVKTTHIPKGWVRG-VNGNVALDYDYALLELKRPHKK 286
PRSS35|Ailuropoda_melanoleuca      229 RRRGKESGRSRRVTEGRPSFQWTRVKNTHIPKGWARG-GKGDAALDYDYALLELKRAHKK 287
PRSS35|Equus_caballus              230 GRRRKGSGRRHRVAEGQPSFQWTRVKNTHIPKGWARG-GSGDAALDYDYALLELKRAHRK 288
PRSS35|Bos_taurus                  229 GRRRKGSARRQRAADGRPSFQWTRVKNTHIPKGWARG-ESRDPALDYDYALLELKRPHKK 287
PRSS35|Sus_scrofa                  231 GRRRNKSARGQRTAAGEPSFQWTRVKHTHIPKGWVRG-ESGDAALDYDYALLELKRSHKK 289
PRSS35|Loxodonta_africana          202 ---------SKRVTEGRPSFQWTRVKNTHIPKGWARG-GRGDAALDYDYALLELKRAHKK 251
PRSS35|Canis_lupus                 229 GRRRKESGRGQRVAEGRPSFQWTRVKNTHIPKGWARG-GKGDAALDYDYALLELKRAHKK 287
PRSS35|Felis_catus                 230 RRRRKESGRGQKAAEGKPSFQWTRVKNTHIPKGWARG-GRGEAALDYDYALLELKRPHKK 288
PRSS35|Mus_musculus                226 QRPGKKSRRGPRVTQGRPSFQWTRVKSTHIPKGWVRG-ENGGLALDYDYALLELKRAHKQ 284
PRSS35|Sarcophilus_harrisii        229 RRRRKESGKGQKGSEGKPSFQWTRVKTTYIPKGWVRG-VRENVALDYDYALLELKRPHKK 287
PRSS35|Rattus_norvegicus           226 QRPGKKSRRGPRVAQGRPSFQWTRVKSTHIPKGWARG-ENGDPALDFDYALLELKRAQKQ 284
PRSS35|Procavia_capensis           230 GRRKKGPGRGQSVFEGRPSFQWTRVKNTHIPKGWVTG-GRGDAALDYDYALLELKRAHRK 288
PRSS35|Sorex_araneus               229 GRRKKESGRGQRVADGRPSFQWTRVKNTHIPKGWARG-GRGEAALDYDYALLELKRAHKK 287
PRSS35|Ictidomys_tridecemlineatus  230 -RGRVELGRGQRVAEGKPSFQWTRVKNTYIPKGWTRG-AGGDAALDYDYALLELKRAHKK 287
PRSS35|Echinops_telfairi           226 GRRRQGRGQGQRAPGGRPSFQWTRVKNTHLPRGWTRG-ARGAAALDYDYALLELKRAHKK 284
PRSS35|Ochotona_princeps           229 RRRRKELGRGQRGADGKPSFQWTRVKNTHIPKGWAK--EGGDPNVDFDYALLELKRAHKK 286
PRSS35|Cavia_porcellus             230 GARQRKPGSGQRGAEGRPSFQWTRVKSIHIPKGWARG-GSGDPALDYDYALLELRRMHKK 288
PRSS35|Mustela_putorius            229 GRRRKESHRGRRVAEGRPSFQWTRVKNTHIPKGWARG-GKGDAALDYDYALLELKRAHKK 287
PRSS35|Oryctolagus_cuniculus       230 SRRRKGSGRGQRVADGRPSFQWTRVKNTHIPKGWARG-QSGDVTLDFDYALLELKRAHKK 288
PRSS35|Erinaceus_europaeus         229 GRR-KESSQGQKVTEGKPSFQWTRVKNMHIPKGWARG-GKGDAALDYDYALLELKRPHKK 286
PRSS35|Myotis_lucifugus            230 ARRRKNSAGGQRGAEGGPSFQWTRVKNTHIPKGWATG-GRGAAALDYDYALLELKRAHKK 288
PRSS35|Meleagris_gallopavo         230 GRKQRRSGRKQASSDGMPSFQWTRVKSTHIPKGWFKG-FSGDIALDYDYAVLELKRPHKR 288
PRSS35|Gallus_gallus               230 GRKQRRSGRKQGSSDGMPSFQWTRVKSTHIPKGWFKG-FSGDVALDYDYAVLELKRPHKR 288
PRSS35|Taeniopygia_guttata         230 ERKQRGSGRKQGTSDGMPSFQWTRVKSTHIPKGWFKG-VSGDIALDYDYAVLELKRPHKR 288
PRSS35|Anolis_carolinensis         233 GRKQKTSGLDEKKTERKPSFQWTRVKSTQIPKGWLRG-VTGDVAVDYDYAVLELKRPHKK 291
PRSS35|Xenopus_tropicalis          238 ELTNNLSGKERGSGAGKPSFQWTRVKAIQIPKGWYRD-VSHNMSLDYDYAVLELKRPHKK 296
PRSS35|Tetraodon_nigroviridis      196 -------------ESEKPSFRWTRVKKSQVPKGWFKG-VSGGLKADYDYAVLELKRPPKV 241
PRSS35|Gasterosteus_aculeatus      199 ----------RSVESGRPLFKWTGVKKTQVPKGWFKG-VSDGLTADYDYAVLELKKAPKV 247
PRSS35|Oryzias_latipes             198 ----------RSVEPNKPSFRWSRVKKTQVPKGWFKG-VSDGLAADFDYAVLELKKAPKM 246
PRSS35|Latimeria_chalumnae         204 --------RSRRSANGKPSFQWTRVKRTQVPKGWFKG-VTDDVAVDYDYAVLELKRPHKQ 254
PRSS35|Ornithorhynchus_anatinus    203 -----------RVVAGKRSFQWTRVKSTQIPKGWVRE-ASGDVVLDYDYAVLELKRPHKK 250
PRSS35|Oreochromis_niloticus       232 KGKSRKSRNRRSVESEKPSFRWTRVKKTQVPKGWFKG-VSGGLAADYDYAVLELKKSSKV 290
PRSS35|Danio_rerio                 235 KGKGRRNRSRRSTDSEQPSFRWTRVKQMQVPKGWFKG-ISENVLADYDYAVLELKRAQKT 293
PRSS35|Gadus_morhua                205 ------------DKADKPSFKWTRVKQTQVPKGWFKGGTSEGVTADYDYAVLELKKAQKV 252
PRSS35|Xiphophorus_maculatus       206 --------SRRSVDSGKPSFKWTRVKKTQVPKGWFKG-VSDGLAADYDYAVLELKRAPKV 256

Conservation
```

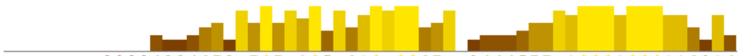

```
--------00004334673+7*7+8*5+69*+**67+-3444577+9***9***996394
```

Supplementary Figure S6D

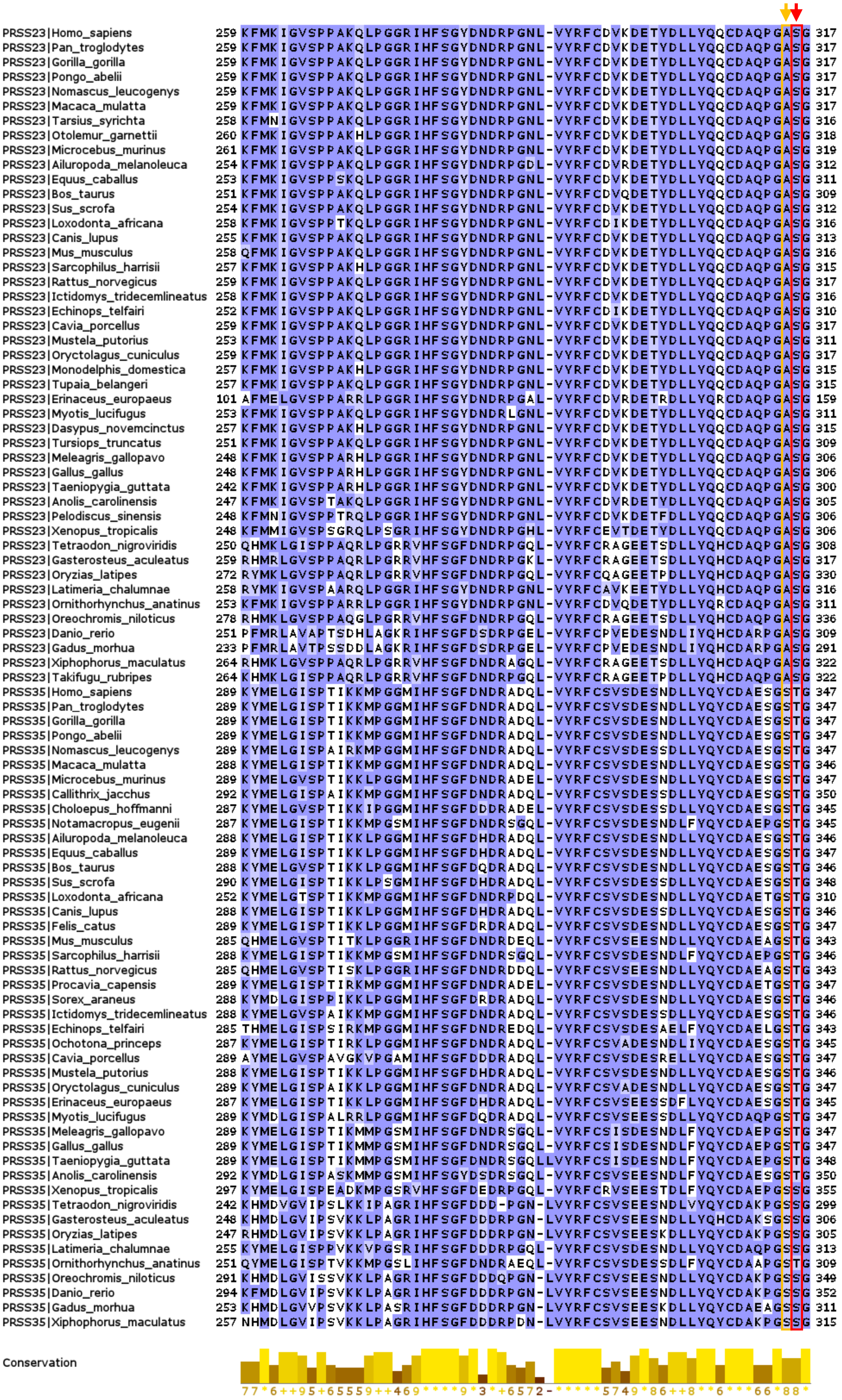

Supplementary Figure S6E

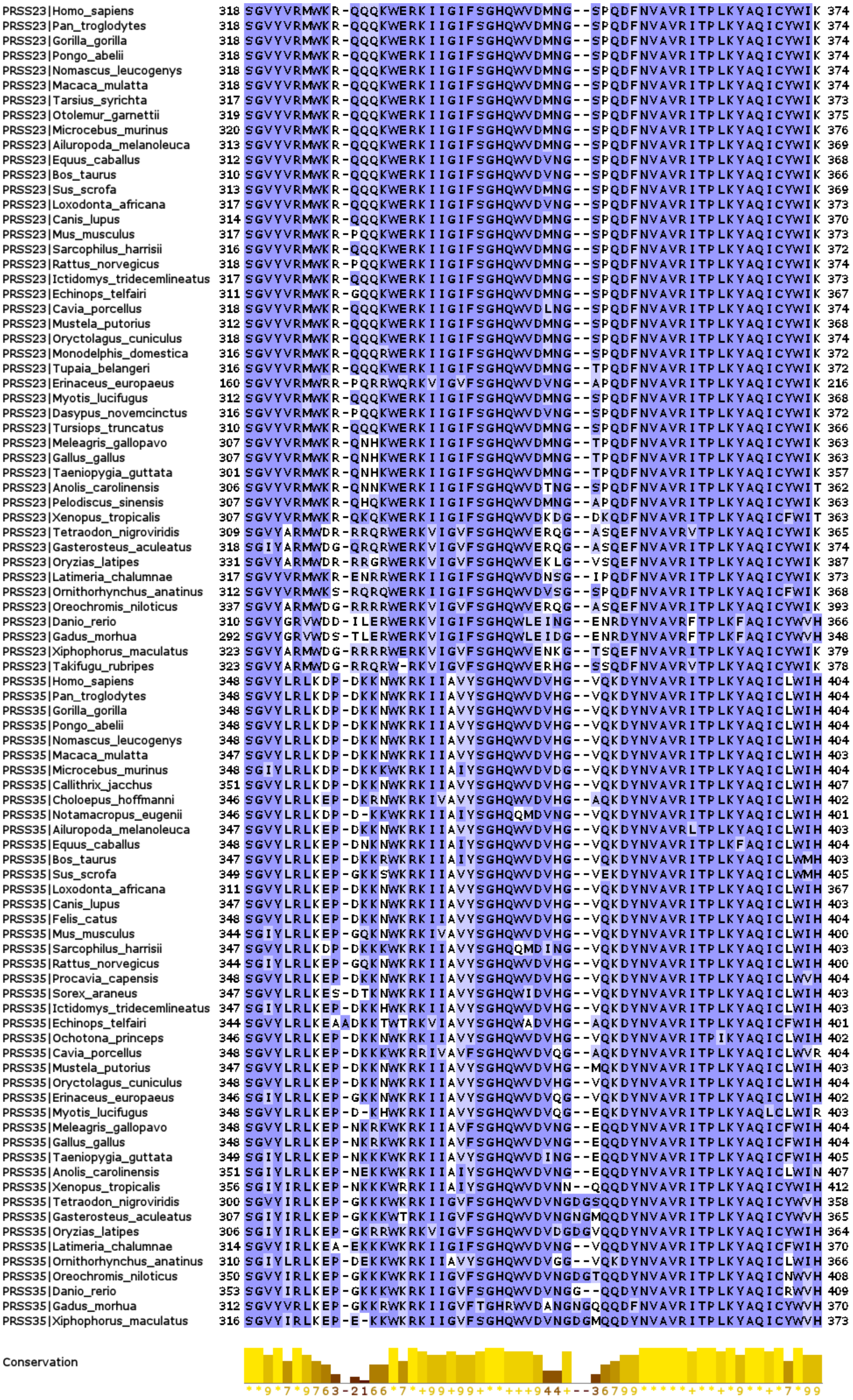

Supplementary Figure S6F

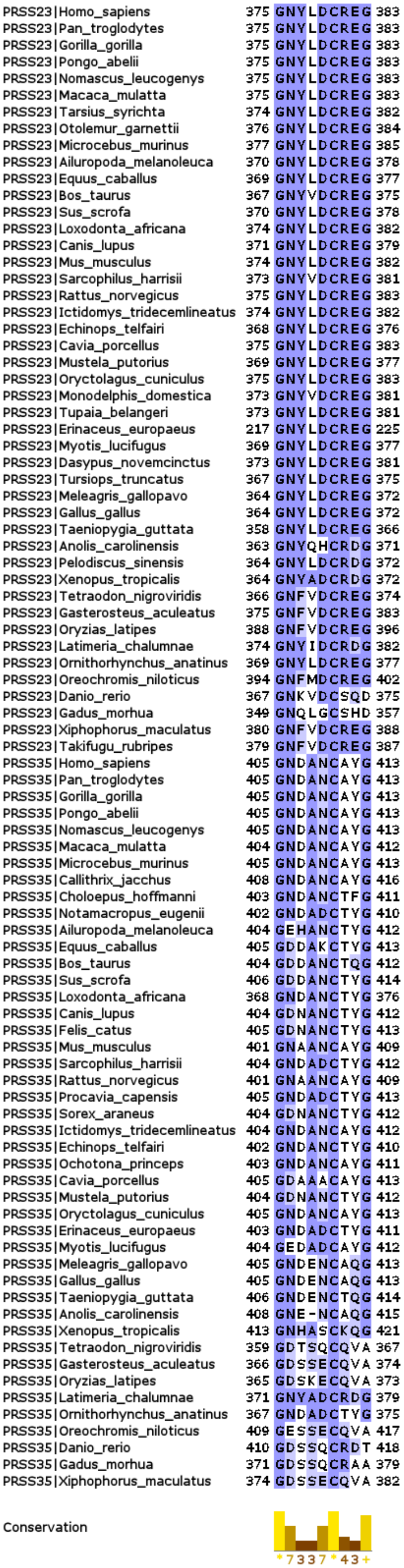

Supplementary Figure S6. Vertebrate PRSS23 and PRSS35 orthologues conserve non-canonical activation-switch residues. *A-F*, Multiple sequence alignment of the PRSS23 and PRSS35 protease domains from vertebrate species (sequences obtained from the MEROPS Database), shown in contiguous segments. *Gold arrows/boxes* indicate the positions corresponding to the predicted mature N-terminus (Ile16-equivalent) and the Asp194-equivalent activation-switch residue; *red arrows/boxes* indicate residues of the catalytic triad. PRSS23 orthologues conserve Gln at the predicted mature N-terminus and Ala at the Asp194-equivalent position. PRSS35 orthologues share the activation-switch substitutions and, in most species, substitute Thr for the catalytic Ser. Alignment is colored by conservation (BLOSUM62), with the conservation histogram shown below each panel. Alignment was generated in Clustal Omega and visualized in JalView

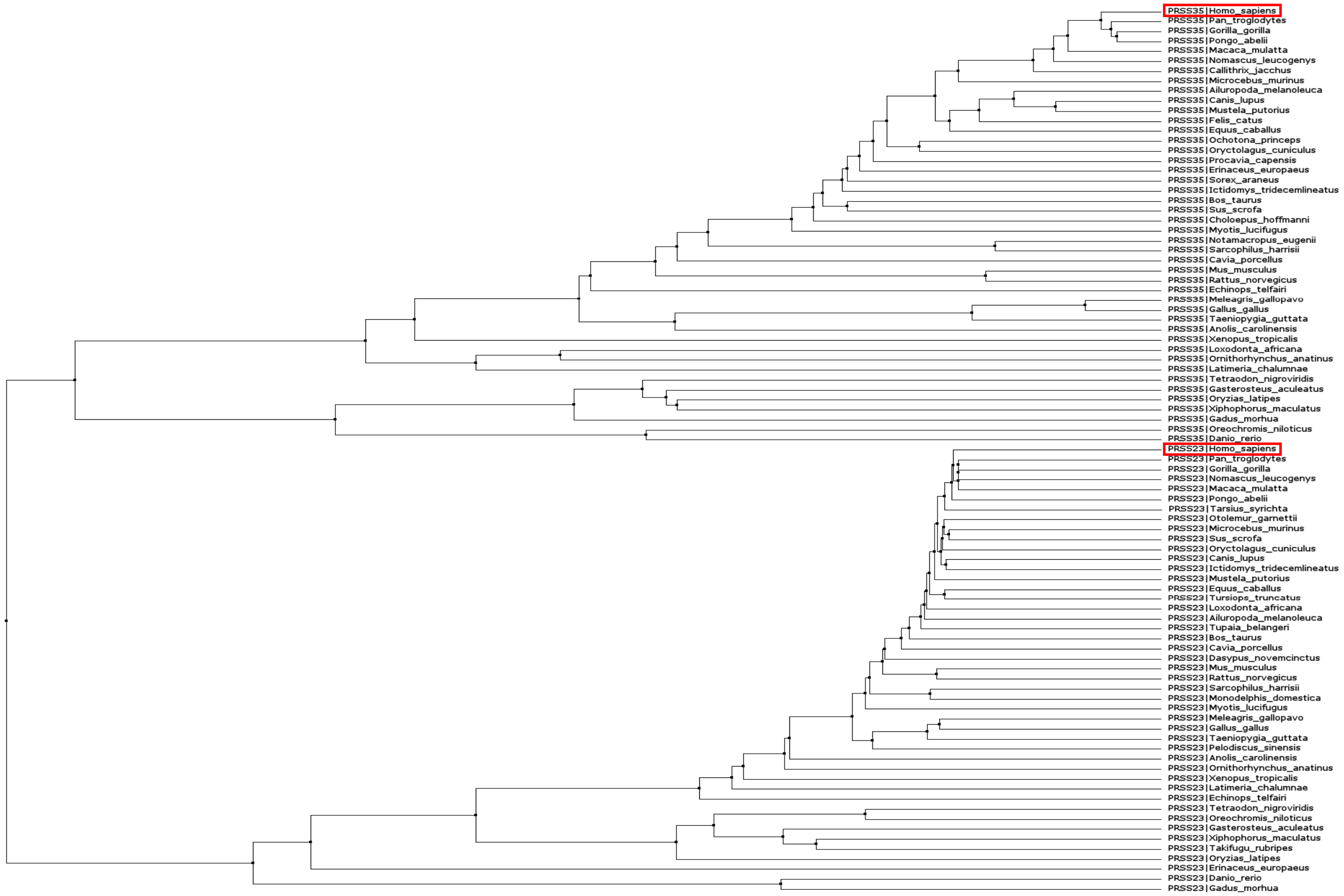

Supplementary Figure S7. Phylogenetic analysis identifies PRSS35 as the closest vertebrate paralogue of PRSS23. Phylogenetic tree inferred from aligned PRSS23 and PRSS35 protease-domain sequences (Supplementary Fig. S6) resolves two major clades corresponding to PRSS23 and PRSS35 orthologues across vertebrates. Human PRSS23 and PRSS35 sequences are highlighted (*boxed*). Tree was generated in JalView from the alignment in Supplementary Fig. S6 using the Average Distance method with a BLOSUM62-based distance matrix.
